# The Missing VP Illusion in Spanish: Assessing the Role of Language Statistics and Working Memory

**DOI:** 10.1162/opmi_a_00118

**Published:** 2024-02-01

**Authors:** Claudia Pañeda, Sol Lago

**Affiliations:** Universidad de Oviedo; Goethe Universität Frankfurt

**Keywords:** missing VP illusion, center-embedding, working memory, language statistics, acceptability judgments, Spanish

## Abstract

In English, double center-embedded sentences yield a so-called “missing VP illusion”: When they are ungrammatical due to a missing verb, they are judged as equally or even more acceptable than their grammatical counterparts. The illusion is often attributed to working memory limitations. Additionally, it has been suggested that statistical differences across languages—e.g., the lower frequency of consecutive verb clusters in verb-initial languages—play a role, since languages with verb-final embedded clauses are less susceptible to the illusion than English. In two speeded acceptability experiments, we demonstrate that the illusion arises in Spanish, a verb-initial language. We also find that the strength of the illusion is modulated by the number of consecutive verbs, consistent with the involvement of language statistics. By contrast, we do not find that participants’ working memory modulates the illusion, failing to support a role of memory limitations. Our results support the generalization that cross-linguistic variation in the missing VP illusion is associated with language statistics and verb position and they demonstrate that this is the case even in languages in which word order is not a reliable processing cue.

## INTRODUCTION

Syntactic research relies on acceptability judgments to uncover speakers’ mental grammar. But empirical judgments sometimes reveal striking dissociations between acceptability and grammaticality, such that grammatical sentences are rejected or ungrammatical sentences are accepted. Such cases have been described as “grammatical illusions” (Phillips et al., 2011). The current study investigates one instance of this, the “missing VP illusion”. Our goal is to help identify the factors that cause a misalignment between acceptability and grammaticality. We do this by testing whether the illusion arises due to the way in which syntactic knowledge interacts with two factors typically described as external to the grammar: human working memory capacity and knowledge about the statistical patterns of a language.

The missing VP illusion occurs in sentences with double center-embeddings (henceforth “double embeddings”). Double embeddings like (1a) are grammatical because center embedding is possible in English and double embeddings simply result from applying this procedure recursively (Chomsky & Miller, 1963). Yet (1a) elicits low acceptability judgments, while the removal of its second verb phrase—which renders the sentence ungrammatical—often improves acceptability. In judgment experiments, English speakers rate sentences like (1b) as equally or even more acceptable than (1a), resulting in a grammatical illusion (Christiansen & MacDonald, 2009; Frank & Ernst, 2019; Frazier, 1985; Gibson & Thomas, 1999). These results are complemented by data showing faster reading times after the final verb when the second verb phrase is missing. This has been interpreted as evidence for eased processing (Frank et al., 2016, 2021; Vasishth et al., 2010). The missing VP illusion has also been found in French acceptability judgments (Gimenes et al., 2009) and a comparable missing noun phrase illusion has been reported in Chinese (Huang & Phillips, 2021).


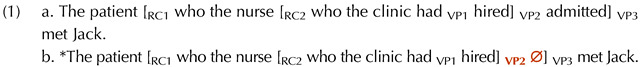


Previous studies have attributed the missing VP illusion to various factors, including constraints in human working memory and language-internal statistics. With regard to working memory, Gibson and Thomas (1999) proposed that each of the three sentence-initial determiner phrases in (1a) creates a prediction of a verb phrase, opening three dependencies that can only be closed after encountering the verbs. Because VP1 is met only after the last determiner phrase, these predictions need to be simultaneously maintained in working memory, which may overload its capacity. The proposal is that the parser tackles the overload by forgetting the VP2 prediction—in Gibson and Thomas’ (1999) framework, the prediction associated with the highest memory cost. As a result, the absence of the VP2 verb in an ungrammatical sentence like (1b) goes unnoticed, yielding a perception of acceptability. In turn, in a grammatical sentence like (1a), the VP2 verb is erroneously attached to the VP3 slot. This causes processing difficulty and unacceptability when the VP3 verb is later encountered, because there is no site left to attach it.^1^

Meanwhile, accounts that link the missing VP illusion to language-internal statistics are motivated by observations of cross-linguistic variation. Specifically, the illusion appears in English, a verb-initial language, but comprehenders of languages with verb-final embedded clauses like German and Dutch are less prone to it, such that grammatical double embeddings like (1a) are accepted more often and read faster than their missing VP counterparts (Bader, 2016; Frank & Ernst, 2019; Frank et al., 2016, 2021; Häussler & Bader, 2015; Vasishth et al., 2010).^2^ To explain this, several accounts grant a role to statistical patterns that are relevant to double embeddings and are proposed to differ in frequency across languages (Christiansen & Chater, 1999; Christiansen & MacDonald, 2009; Engelmann & Vasishth, 2009; Frank & Ernst, 2019; Frank et al., 2016, 2021; Futrell et al., 2020; Futrell & Levy, 2017).

One such pattern is represented by consecutive verbs sequences. Grammatical double embeddings like (1) contain three consecutive verbs. In English, it has been proposed that the illusion is associated to the infrequency of such configurations, which makes them unexpected. By contrast, removing one VP leads to a pattern of two consecutive verbs, which is more frequent and expected and thus favors the illusion (Frank & Ernst, 2019; Frank et al., 2016, 2021).

The fact that German and Dutch speakers are less prone to the illusion is attributed to three-verb sequences being more frequent in these languages, due to the verb-finality of embedded clauses (Frank et al., 2016; Vasishth et al., 2010). Verb-finality favors the appearance of verb clusters at the end of a clause, which may in turn precede a main verb, as in the German sentence *Auch ihre Augen* [_RC_ *die schon vieles gesehen haben mochten*] *schienen dem grässlichen Bann seines Schmerzes verfallen zu sein* (‘Even her eyes, which might have already seen many things, seemed to have fallen under the ghastly spell of his pain’), retrieved from German Web 2020 (deTenTen, 2020; Kilgarriff et al., 2004, 2014). In support of this account, neural network surprisal models have shown that differences between English and German/Dutch—learned from toy languages based on them and corpora—predict the cross-linguistic contrast in the missing VP illusion. In English, these models predict higher acceptability ratings and/or lower surprisal values and thus more processing ease in double embeddings with a missing VP, whereas the opposite prediction is obtained for German and Dutch (Christiansen & MacDonald, 2009; Engelmann & Vasishth, 2009; Frank et al., 2016; see also Futrell et al., 2020; Futrell & Levy, 2017).

In short, previous research suggests that both working memory capacity and knowledge about the frequency of consecutive verbs may influence the missing VP illusion. The current study extends this research by assessing the relevance of these factors in the comprehension of Spanish, a language in which the missing VP illusion has only been tested in production (Hahn et al., 2022). We do this in two experiments that measure the illusion with a speeded acceptability judgment task. Experiment 1 evaluates the role of consecutive verb patterns by capitalizing on a property of Spanish, subject-verb inversion, which allows manipulating the number of consecutive verbs while keeping the number of embeddings and the grammatical status of the sentences constant. Experiment 2 assesses the role of working memory by examining whether Spanish participants’ working memory predicts their susceptibility to the illusion. Before presenting the experiments, we describe the properties of Spanish that distinguish it from previously studied languages.

### Properties of Spanish Relevant for the Missing VP Illusion

This study investigates cross-linguistic variation in the missing VP illusion by providing data from Spanish. To date, cross-linguistic differences in the missing VP illusion have been associated with word order: Languages which show the illusion are verb-initial, whereas languages that are more resilient to the illusion have verb-final embedded clauses. From this perspective, we may expect Spanish to show the illusion, because it is a verb-initial language. However, evidence that verb-initial languages exhibit the illusion comes mostly from English, a language in which word order is rather fixed and thus provides a reliable cue for parsing (MacWhinney et al., 1984).^3^ In contrast, Spanish has freer word order and thus Spanish speakers may be less reliant on word order and more on other cues, such as inflectional agreement (Bornkessel-Schlesewsky et al., 2011; Bornkessel-Schlesewsky & Schlesewsky, 2009; Bornkessel & Schlesewsky, 2006; Kail, 1989; MacWhinney, 1987; MacWhinney et al., 1984; Norcliffe et al., 2015). This cross-linguistic contrast could lead to different processing strategies for double embeddings in Spanish vs. English comprehenders.

Cross-linguistic differences could also arise due to other differences between Spanish and English. For instance, Spanish exhibits differential object marking, such that animate and specific objects are typically introduced by the preposition *a* (Fábregas, 2013; Leonetti, 2003; Torrego, 1998). In the Spanish equivalent of double embeddings like (1), this preposition can precede the relative pronoun, as shown in (2) (the relevant prepositions, amalgamated with the article *el* in the form *al*, are bolded). The preposition signals to comprehenders that the relative pronoun has a non-subject role in the relative clause. This could facilitate processing in at least two ways. First, it could help with the assignment of thematic roles, which may be particularly difficult due to the noncanonical object-subject order (see Lau & Tanaka, 2021 for a review of the subject relative clause advantage). Second, it could cause comprehenders to commit to an object relative clause analysis as soon as the relative pronoun is encountered, reducing the probability that they initially misparse it as a subject relative clause and have to reanalyze later (del Río et al., 2012).


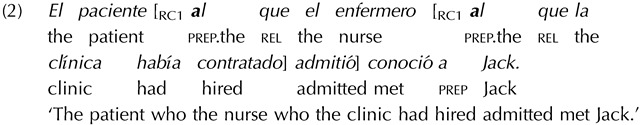


An eased processing of double embeddings in Spanish could make the missing VP illusion less likely in Spanish than in English. This possibility is supported by a self-paced reading study that measured reading times and comprehension accuracy in sentences with center-embedded vs. right-branching clauses in Spanish and English (Hoover, 1992). We focus here on the comprehension accuracy of center-embedded sentences, because reading times were only analyzed to compare center-embedding with right-branching structures, which are irrelevant for our purposes. On average, English and Spanish speakers were about 85% accurate in their comprehension of single embeddings, but a clear cross-linguistic difference obtained with double embeddings: While English speakers’ accuracy dropped to about 40%, Spanish speakers were still about 85% accurate. This supports an easier comprehension of double embeddings in Spanish. Since Hoover (1992) did not test double embeddings with a missing second verb, it is unknown whether the missing VP illusion occurs in Spanish comprehension. However, recent findings from production suggest that this might be the case. In a sentence completion task, Hahn et al. (2022) found that Spanish speakers completed fragments with double embeddings—e.g., *the fact that the director who*—with two rather than the three required verbs at rates that ranged from 30% to 70%. Further, the percentage of missing verbs was higher when the first noun (e.g., *fact*) had a lower embedding bias—i.e., a lower log-probability to be followed by *that* and thus to introduce an embedded clause—, suggesting a role for speakers’ knowledge about the statistics of their language. The current study complements these findings from production by examining whether Spanish speakers fail to notice missing verbs in the comprehension of double embeddings.

### Assessing the Role of Working Memory

Another goal of our study is to assess whether the missing VP illusion is related to limitations in comprehenders’ working memory capacity. Many accounts have proposed this, but to date it has not been directly tested, for example by examining whether the size of the illusion is influenced by an independent measure of comprehenders’ working memory capacity. To assess this, we obtained a quantitative measure of working memory capacity via an operation span task (Turner & Engle, 1989). In this task, participants need to remember letters while verifying mathematical operations, and their working memory capacity is scored based on the rate of correctly recalled letters: Higher recall rates are indicative of greater capacity. If working memory capacity predicts the size of the illusion, we expect comprehenders with higher working memory scores to be less prone to the illusion. This is because higher capacity speakers are known to process complex material such as sentences with object relative clauses and center-embeddings more easily and successfully than their lower capacity peers (Chen et al., 2008; Fiebach et al., 2004; King & Just, 1991; Prat et al., 2007; Vos & Friederici, 2003; Vos et al., 2001). Thus, speakers with a higher memory capacity should be more able to process double embeddings and to detect missing verbs, reducing the missing VP illusion as compared to comprehenders with lower working memory spans. Given our interest in working memory, we measured the illusion with a speeded acceptability judgment task, in which sentences are presented word by word and judged under time pressure. We chose a speeded task because it is more likely than an untimed task to tap into working memory differences, since there is little time to reflect on the acceptability intuitions and sentences cannot be read multiple times (Drenhaus et al., 2005; Parker & Phillips, 2016; Wagers et al., 2009).

### Assessing the Role of Language Statistics

A second goal of our study is to assess whether the missing VP illusion is modulated by comprehenders’ knowledge of the statistical patterns of their language, specifically the frequency of consecutive verb clusters. To date, the role of language statistics in the missing VP illusion has been assessed with cross-linguistic comparisons, by comparing how susceptible to the illusion are speakers of SVO (subject-verb-object) vs. SOV (subject-object-verb) languages, who should differ in their exposure to verb final structures and consecutive verb patterns. An alternative way to test the role of language statistics is through a within-language manipulation. We do this using subject-verb inversion in Spanish, which allows us to vary the order of the subject and the verb in the most embedded relative clause: subject-verb (SV) in (3a) vs verb-subject (VS) in (3b).


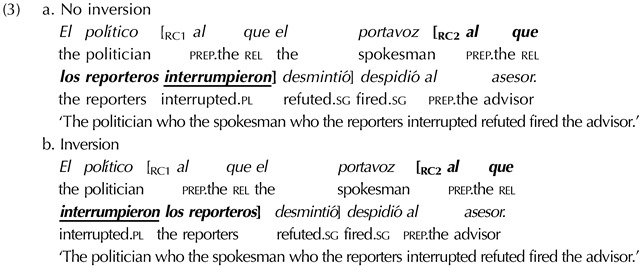


Both the SV and the VS order are grammatical in Spanish. SV is usually the neutral or unmarked order in simple declarative sentences, but VS may also be unmarked in several circumstances, for instance, if the subject is bare—not preceded by a determiner or quantifier—, or if the verb is unaccusative (*Llegaron niños* ‘(Some) children arrived’), psychological (*Me gustan las películas de Billy Wilder* ‘I like Billy Wilder’s movies’) or in the infinitive form (*Al salir el sol* ‘When the sun rises’, literally ‘At the rising.inf of the sun’) (Olarrea, 2012). In other configurations, the VS order is used in marked contexts, notably when the subject is focused (Olarrea, 2012; Ordóñez, 2000; Zubizarreta, 1998). Furthermore, the VS order is typically observed—and appears to be obligatory in European Spanish—in syntactic configurations like wh-questions and exclamatives (Francom, 2012). In relative clauses, which are the focus of our study, both orders are possible, with VS being unmarked and SV typically being used for subject topicalization (Gutiérrez-Bravo, 2013). Subject position can also be affected by semantic factors, like agentivity and definiteness, with a tendency towards postposing non-agentive and non-definite subjects (López Meirama, 2006).

For our study, the relevant point is that subject-verb inversion reduces the number of consecutive verbs in sentences with double embeddings. Language statistics accounts propose that the illusion occurs in verb-initial languages because comprehenders expect two rather than three consecutive verbs, even if the sentence is ungrammatical with just two verbs. If this account is on the right track, a similar difference might be expected between double embeddings that differ in the number of consecutive verbs due to inversion, like (3a) and (3b): Inversion should help comprehenders’ judge double embeddings in a way that aligns with their grammatical status, reducing the missing VP illusion.

Crucially, whether a reduction in the number of consecutive verbs should increase the acceptability of grammatical double embeddings depends on whether this yields a more frequent configuration: Center-embeddings with inversion—and thus fewer consecutive verbs—should be more frequent than center-embeddings without inversion—and thus more consecutive verbs. To get an idea of whether this is the case in Spanish, we conducted a preliminary corpus search. Note that this was not a formal corpus study, because it was limited in scope to constructions similar to our experimental materials (see below). The results of the corpus search were used to motivate experimental predictions that were then formally evaluated in Experiment 1.

The search was conducted in the Corpus del Español del Siglo XXI (CORPES XXI) (Real Academia Española, 2022). CORPES XXI is a collection of written and oral Spanish texts starting in the year 2001. It contains about 350 million tokens. Our search was restricted to texts from the 2010–2022 period that were produced in Spain, the country of origin of our participants. Both written and oral texts were included (for more details, see Appendix A).

Our goal was to compare the frequency of the structures underlying (3a) and (3b), shown in (4a) and (4b). Specifically, we looked for sentences with two center-embedded relative clauses introduced by the relative pronoun *que* (‘that’) that met the following conditions: (a) Each relative pronoun had a determiner phrase antecedent that was the subject of the nearest outer verb, and (b) the inner relative clause only comprised a subject and a verb presented in either a subject-verb order (4a), with three consecutive verbs, or a verb-subject order (4b), with two consecutive verbs.

(4)  a. S_i_ [_RC1_ *that*_i_ S_j_ [_RC2_ *that*_j_ S V] V] V
    b. S_i_ [_RC1_ *that*_i_ S_j_ [_RC2_ *that*_j_ V S] V] V

Our goal was to see whether structures like (4b) were more frequent than structures like (4a). However, we could not find any instance of double embeddings, either with or without inversion. This suggests that double center embeddings—at least those with the properties that we selected—are extremely infrequent in European Spanish. At first sight, this might predict that inversion should not modulate the acceptability of double embeddings: If comprehenders have little-to-no exposure to either inverted or non-inverted double embeddings, they should not have a preference for either option and they may reject both at the same rate. However, we hypothesized that, if comprehenders have a preference for two vs. three consecutive verb patterns in other grammatical contexts, they might still be influenced by this preference when processing and judging double embeddings.

To assess whether there is such a preference, we compared the number of cases with and without inversion in related sentences that were indeed attested in the corpus—sentences with single center-embeddings, which have a parallel structure with just one level of subordination. An example of the structures we examined is shown in (5): Single embeddings without inversion (5a) had two consecutive verbs and single embeddings with inversion (5b) had no consecutive verbs (for each structure, we show an attested sentence).

(5)  a. S_i_ [_RC_ *que*_i_ S V] V
     *El tipo de habilidades que las empresas*_.S_ *requieren*_.V_ *seguirá cambiando*
     ‘The type of skills that companies require will continue to change’
    b. S_i_ [_RC_ *que*_i_ V S] V
     *Los primeros presupuestos generales que apruebe*_.V_ *el Gobierno*_.S_ *corresponderán ya al ejercicio 2021*
     ‘The first general budget that the Government will approve will already correspond to the fiscal year 2021’

Numerically, single embeddings were more frequent with inversion than without inversion: 436 vs. 32 tokens. We hypothesized that this higher frequency might facilitate processing and increase acceptability both in single embeddings and double embeddings. If so, inversion should increase our participants’ ability to properly detect the grammaticality status of double embeddings, thus favoring a closer alignment between acceptability and grammaticality and reducing the missing VP illusion.

## EXPERIMENT 1: THE ROLE OF LANGUAGE STATISTICS

Experiment 1 investigated whether the missing VP illusion occurs in Spanish and whether it is modulated by inversion and its reduction of consecutive verb clusters. With this goal, two factors were manipulated: the grammaticality of the sentence (grammatical vs. ungrammatical) and subject-verb inversion in the second embedded clause (non-inverted vs. inverted). Grammatical conditions contained all three verb phrases, while ungrammatical conditions had a missing VP2 (Table 1). Non-inverted conditions had a subject-verb order in the second embedded clause (e.g., ‘the reporters._S_ interrupted._V_’), whereas the inverted conditions had verb-subject order (e.g., ‘interrupted._V_ the reporters._S_’).

**Table 1.**
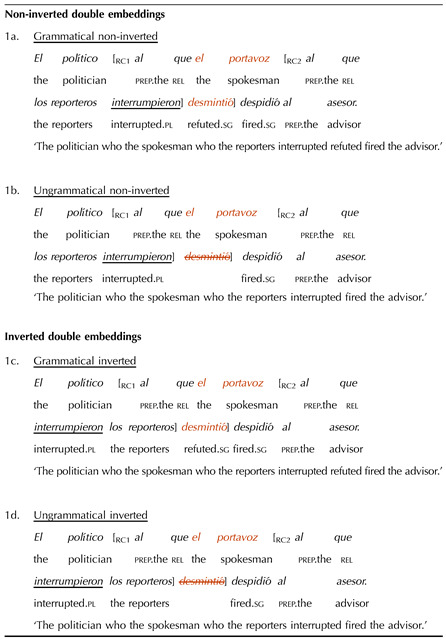
Sample double center-embedded item in Experiment 1

*Note*. The critical verb (VP2) is crossed-out in the ungrammatical conditions to represent its omission. The verbs whose position was manipulated are underlined.

Our predictions were as follows: If the missing VP illusion occurs in Spanish, ungrammatical double embeddings should be more acceptable than grammatical double embeddings. Further, if consecutive verb patterns contribute to the illusion, subject-verb inversion should reduce its size, as it reduces the number of consecutive verbs and this should yield a more frequent configuration. The illusion could be reduced by an increase in the acceptability of the grammatical conditions, where participants should be more able to perceive that the sentences are well-formed. It could also be reduced by a decrease in the acceptability of the ungrammatical conditions, where participants should be more able to detect the missing VP.

### Method

#### Participants.

Eighty-one self-reported native speakers of European Spanish were recruited through Prolific (2023, www.prolific.co) and participated in the experiment. To reduce the likelihood of including second language speakers in our analysis, participants completed a Spanish proficiency test after the experiments, which resulted in the exclusion of 4 participants with less than 90% accuracy. The remaining 77 participants had a mean age of 29 years (range 18–49) and no self-reported language impairments. Thirty-seven self-identified as female, 39 as male and 1 as non-binary. Six were left-handed. In this and the following experiment, participants provided informed consent and all procedures were in accordance with the Declaration of Helsinki.

#### Materials.

Experimental materials comprised 24 double embedding item sets (Table 1; full set of materials available in Appendix B). Embedded clauses were relative clauses introduced by the preposition *a*, which unambiguously signaled them as object relative clauses. As a result, their antecedent could not be grammatically interpreted as the subject of the clause-internal verb, not even in the conditions with inversion, in which the verb appeared before its subject. In these clauses, the interpretation of the determiner phrase following the verb as its subject was further ensured through plural agreement (the subject-verb dependencies in the other clauses were always singular).

Twelve single embedding item sets were included as a control manipulation. This was to ensure that participants’ potential acceptance of double embeddings with missing VPs was not due to length differences. Note that, because of the missing verb, the missing VP conditions were always shorter than the grammatical conditions. This shorter length could make them easier to process and thus perceived as more acceptable. We reasoned that if this was the case, participants should also apply this “length heuristic” to single embeddings. The single embeddings had the same missing VP manipulation as the double embeddings, but in a single-clause configuration, which should be easier to process and thus not overload memory resources. Therefore, if participants’ performance was driven by memory limitations, they should show an illusion of grammaticality with double but not with single embeddings, with the latter triggering a canonical grammaticality effect.

To ensure comparability with the experimental items, half of the single embedded items featured subject-verb inversion, while the other half did not. Further, half of the items had a plural subject in the embedded clause, while the other half had a singular subject. A sample single embedded item set is shown in (6). (The critical verb is crossed-out in the ungrammatical condition to represent its omission.)


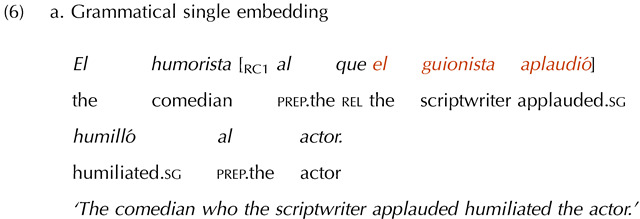



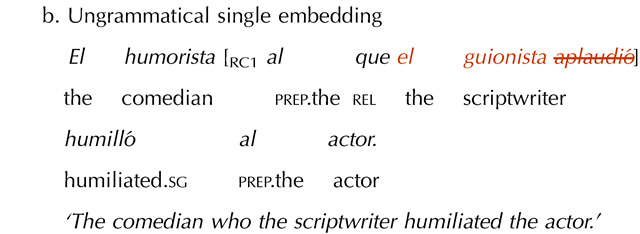


#### Procedure.

Participants performed the speeded acceptability judgment task online in IbexFarm (Drummond, 2013). After completing a demographic questionnaire, the participants read the experiment instructions, which indicated that they had to rate the acceptability of the sentences at a fast pace, basing their judgments on intuition rather than on academic norms, plausibility or sentence length. After 4 practice trials, they carried out the acceptability task. Sentences appeared word-by-word at a fixed rate of 400 ms per word. After the sentence, participants had 2000 ms to judge its acceptability by pressing the keys *J* (‘acceptable’, right hand) or *F* (‘unacceptable’, left hand). If they failed to respond within the deadline, they were shown the message *Too late!* and were instructed to proceed to the next screen.

Single and double embeddings were intermixed with 72 fillers (half ungrammatical). The order of presentation of filler and experimental items was pseudo-randomized for each participant, such that sentences from the same condition never appeared consecutively. Experimental items were distributed across four Latin square lists, such that each participant only saw one condition of each experimental sentence.

At the end of the experiment, there was a multiple-choice grammar test consisting of 10 sentences, which was used to verify our participants’ knowledge of Spanish. Based on pilot testing, an accuracy of 90% or above was established for a participant to be included in the analysis. Participants took on average 27 minutes to complete the experiment (range across participants: 18–71 minutes).

#### Analysis.

Acceptability judgments were coded as 0 (unacceptable) and 1 (acceptable) and analyzed with mixed-effects logistic regression (Barr, 2008; Quené & van den Bergh, 2008). Responses with reaction times shorter than 100 ms or longer than 2000 ms were excluded: This affected approximately 3.14% of the data (*SD* = 3.10%). Response latencies were not analyzed for two reasons. First, because positive (acceptance) and negative (rejection) responses typically elicit different response time patterns (Ratcliff, 1985). Second, because acceptable and unacceptable responses were given with different hands and thus confounded with laterality: Longer latencies in ungrammatical sentences could be due either to processing disruptions or to a left-hand disadvantage (most participants were right-handed).

The analysis was implemented with Bayesian models using the brms package in R (Bürkner, 2017; R Core Team, 2023). Bayesian models are useful because they combine prior information with evidence from the data in order to obtain a probability distribution over the plausible values of a parameter—the parameter’s posterior distribution. Thus, an experimental effect can be quantified in terms of the likelihood of its possible values, which can be more informative than a binary significance statement because it puts the focus on determining an effect size and its direction, along with its uncertainty (Cumming, 2014; Kruschke & Liddell, 2018).

Two models were used to quantify the existence of a missing VP effect and its modulation by subject-verb inversion. The first model directly compared single and double embeddings and assessed whether there was a missing VP illusion in double embeddings. All fixed effects were sum-coded, as specified between parentheses (the baseline level is listed first).

The fixed effects of the first model were grammaticality (−0.5 grammatical / 0.5 ungrammatical), number of embeddings (−0.5 single / 0.5 double) and their interaction. If a missing VP effect occurs in Spanish, there should be a grammaticality × number of embeddings interaction, supporting increased acceptability for ungrammatical vs. grammatical sentences with double embeddings, but the opposite pattern for single embeddings (a canonical grammaticality effect).

The second model focused on double embeddings to quantify the effect of subject-verb inversion. The fixed effects were grammaticality (−0.5 grammatical / 0.5 ungrammatical), inversion (−0.5 non-inversion / 0.5 inversion) and their interaction. If inversion affects the missing VP illusion, there should be an inversion × grammaticality interaction indicating a smaller missing VP illusion in sentences with inversion.

We used weakly informative priors to constrain the models to psycholinguistically plausible parameter estimates while ensuring that the priors would not outweigh the evidence provided by the data (Gelman et al., 2008). Specifically, a standard normal distribution N(0, 1) was used for all fixed effects except for the intercept, which had a N(0, 10) prior. The prior for the standard deviation of the random effects and for the residual standard deviation used a half-normal distribution N^+^(0, 1), because these parameters cannot be negative. Within the variance-covariance matrices of the by-participant and by-item random effects, priors were defined for the correlation matrices using a Lewandowski-Kurowicka-Joe prior (Lewandowski et al., 2009). This prior has a parameter *η*, which, when set to 2, has the regularizing effect of disfavouring extreme correlations. All models had a maximal random effects structure, with correlated varying intercepts and slopes for items and participants for all main effects and their interactions (Barr et al., 2013). All data and code necessary to reproduce our results are available at https://osf.io/kp8ns/.

### Results

**Figure 1.**
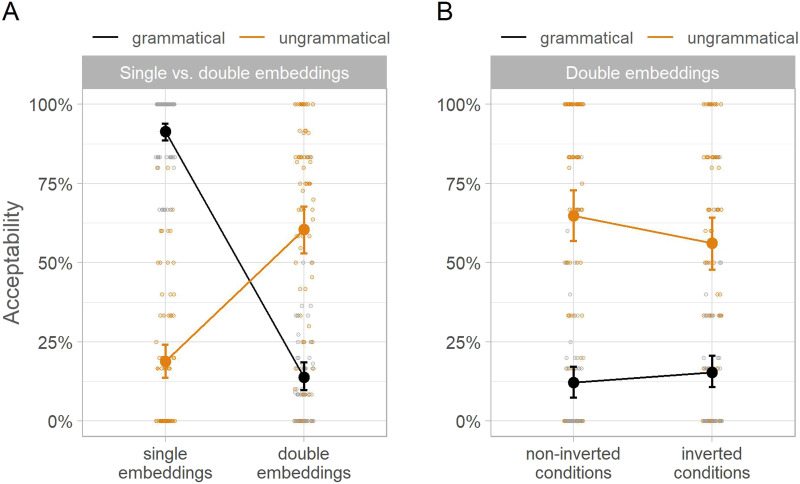
Acceptance rates in Experiment 1. *Note*. Panel A illustrates the missing VP effect in the empirical acceptance rates: Ungrammatical double embeddings were accepted more often than grammatical ones, while the opposite was true of single embeddings. Panel B illustrates the effect of subject-verb inversion in double embeddings: The missing VP effect was smaller in inverted vs. non-inverted conditions. Larger points show empirical condition means across participants and error bars show 95% bootstrapped confidence intervals. Smaller points depict by-participant means.

Accuracy in the filler items was high, showing that participants could cope well with the speeded presentation rate (mean accuracy = 94%, range across participants: 72–100%). Figure 1 shows a descriptive summary of the empirical acceptance rates of the experimental conditions. Table 2 shows the model estimates, with their mean and 95% credible interval (CrI), which is the interval in which the true mean is estimated to lie with 95% probability. Statistical analyses and inferences were always based on those estimates, but the text reports them back-transformed to percentages for easier interpretability.

**Table 2.** Model estimates in Experiment 1

	Estimate	95% CrI
**Missing VP illusion model**		
Intercept (grand mean)	−0.28	[−0.60, 0.04]
Number of embeddings	−1.21	[−1.64, −0.79]
Grammaticality	−0.68	[−1.10, −0.26]
Number of embeddings × Grammaticality	6.65	[5.73, 7.48]

**Inversion model**		
Intercept (grand mean)	−1.07	[−1.57, −0.60]
Inversion	−0.07	[−0.44, 0.32]
Grammaticality	3.13	[2.49, 3.76]
Inversion × Grammaticality	−1.10	[−1.80, −0.41]

*Note*. Columns show the estimated effect size (Estimate) and its 95% credible interval (CrI) in log odds. On the log odds scale, a value of zero corresponds to 50% acceptability. For the factor Number of embeddings, a negative coefficient indicates lower acceptability in double embeddings. For Grammaticality, a negative coefficient indicates lower acceptability in ungrammatical sentences. For Inversion, a negative coefficient indicates lower acceptability in sentences with subject-verb inversion.

#### The Missing VP Effect.

The comparison of single and double embeddings revealed a grammaticality × number of embeddings interaction with a posterior mean of 93% and a 95% CrI of [89, 95] %. This interaction indicated opposite grammaticality effects for sentences with single vs. double embeddings. With single embeddings, ungrammatical sentences were accepted less often than grammatical sentences, consistent with a canonical grammaticality effect: −63 [−72, −52] %. By contrast, the pattern was reversed in double embeddings: Ungrammatical sentences were accepted more often than grammatical sentences, consistent with a missing VP effect: 30 [20, 40] %.

#### The Effect of Subject-Verb Inversion.

The comparison of double embeddings with and without inversion revealed a grammaticality × inversion interaction: −21 [−34, −7] %. Consistent with the predictions of language statistics accounts, the interaction arose due to a smaller missing VP effect in inverted vs. non-inverted conditions: 45 [31, 57] % vs. 65 [49, 81] %. The change in the missing VP effect size was driven by the ungrammatical conditions: Inversion reduced the acceptance of ungrammatical sentences (−12 [−21, −3] %), but it did not have a consistent effect in grammatical sentences, with a 95% credible interval that spanned positive and negative numbers: 9 [−2, 20] %.

### Discussion

Experiment 1 found that Spanish speakers accepted ungrammatical double embeddings more often than grammatical double embeddings. This demonstrates a missing VP illusion in Spanish. We also found that subject-verb inversion modulated the illusion: While ungrammatical sentences were overall more acceptable than grammatical sentences, the difference was smaller when the most embedded clause featured subject-verb inversion. Since inversion reduces the number of consecutive verbs, this finding supports the claim that consecutive verb patterns contribute to the illusion, as claimed by language statistics accounts (Christiansen & Chater, 1999; Christiansen & MacDonald, 2009; Engelmann & Vasishth, 2009; Frank & Ernst, 2019; Frank et al., 2016, 2021; Futrell et al., 2020; Futrell & Levy, 2017). These accounts attribute the role of consecutive verb patterns to their infrequency, which makes them less expected and thus difficult to parse. The inversion effect may thus be caused by the higher frequency of the configuration.

Note, however, that we had a priori assumed that inversion would help comprehenders correctly reject ungrammatical double embeddings and also accept grammatical double embeddings. Our results numerically aligned with these predictions, but the effect of inversion was only reliable in ungrammatical sentences. How might this be explained? One possibility relates to how inversion affected the number of consecutive verbs in each case. In the ungrammatical conditions, inversion reduced the number of consecutive verbs from two to zero. According to our corpus search, zero consecutive verbs are more frequent than two consecutive verbs in center-embedded sentences. Thus, inversion in the ungrammatical conditions clearly increased the frequency of the structure, which may have facilitated processing enough for participants to detect the ungrammaticality more often.

Meanwhile, in the grammatical conditions, inversion reduced the number of consecutive verbs from three to two. While two consecutive verb sequences are probably more frequent than three-consecutive-verb sequences—which we did not find in the corpus search—two consecutive verb sequences are still very infrequent. Because of this, inversion in grammatical double embeddings may not have facilitated processing enough to improve participants’ ability to judge grammatical sentences as acceptable.

Before proceeding to the next section, we consider two alternative interpretations of the subject-verb inversion effect that do not rely on the reduction of the number of consecutive verbs, and therefore do not necessarily support a role of language statistics in the missing VP illusion. First, inversion may have attenuated the illusion because it is the preferred option in relative clauses, perhaps because it is unmarked (Gutiérrez-Bravo, 2013). This could have made sentences with inversion more natural than sentences without inversion, which are used for subject topicalization and may be less felicitous in the absence of a motivating context. While this is a viable alternative explanation, we find it less likely. Word order preferences depend on multiple factors, which can have opposite effects. For example, even if inversion was preferred for syntactic or pragmatic reasons, it may have been dispreferred for semantic reasons, since our subjects were agentive and definite and the tendency is for non-agentive and non-definite subjects to appear in postverbal positions (López Meirama, 2006). In addition, in the single embedding conditions, grammatical sentences with and without inversion were rated very similarly (Mean_NON-INVERTED_ = 89%, *SD*_NON-INVERTED_ = 24; Mean_INVERTED_ = 93%, *SD*_INVERTED_ = 15). This suggests that non-inverted sentences were highly acceptable and not strongly dispreferred compared to their inverted counterparts.

A second alternative interpretation is that inversion weakened the illusion because it reduced comprehenders’ memory load. This could be argued, for instance, in the framework of Dependency Locality Theory (Gibson, 2000). Dependency Locality Theory provides a metric for computing the cost of integrating the members of a dependency, like the one between the pronoun (a)*l que* (‘that’) and the object gap following the verb *interrupted* (e.g., *al que los reporteros interrumpieron __*; Table 1). The metric is based on the number of discourse referents between the two members of the dependency. In non-inverted conditions, the two members of the dependency are separated by two discourse referents, the subject noun and the verb (e.g., *al que los reporteros interrumpieron __*). But in the inverted conditions, the two members of the dependency could be separated by only one discourse referent, the verb (e.g., *al que interrumpieron __ los reporteros*). As a result, the inverted conditions might have a lower integration cost and cause less memory load. The same contrast would arise if the dependency was resolved directly at the verb, but with one less intervening discourse referent in each case.

Under this view, the inversion effect would support a role of memory in the missing VP illusion, rather than a role of language statistics. While this is a viable interpretation, we note that it depends on specific assumptions that may vary depending on the theoretical framework, e.g., assumptions about the relationship between discourse referents and memory load and/or about the location of the gap. For example, regarding the gap position, the difference between inverted and non-inverted conditions is only predicted if the gap is located between the verb and the subject in the inverted conditions. This may be the case if the verb-subject order results from a movement of the subject to the right of the VP (e.g., Rizzi, 1982). However, if the verb-subject order arises because the subject remains in situ and the verb moves to a higher position (e.g., Ordóñez, 2000), the gap would be located after the subject (e.g., *al que interrumpieron los reporteros __*). As a result, the two members of the dependency would be separated by the same number of discourse referents in the inverted conditions and non-inverted conditions, and thus, the inversion effect would not support memory accounts anymore. Due to these issues, we favor an interpretation of the effect of inversion in terms of language statistics. In any case, such an interpretation does not rule out the possibility that memory limitations additionally contribute to the illusion. We address the role of memory in Experiment 2.

## EXPERIMENT 2: THE ROLE OF WORKING MEMORY

Experiment 2 sought to replicate the missing VP illusion and to investigate whether it was affected by limitations in participants’ working memory capacity. To measure the missing VP illusion, we again compared single and double embeddings. To assess the effect of working memory, we examined whether the size of the illusion was modulated by the participants’ working memory scores, independently measured in an operation span task. If the illusion is affected by working memory limitations, individuals with higher working memory capacity should be less likely to forget verb predictions (Gibson & Thomas, 1999) or to suffer from retrieval interference (Bader, 2016; Häussler & Bader, 2015) than their low-capacity peers. Thus, individuals with higher working memory capacity should be less prone to the illusion. Finally, a working memory effect should appear with double embeddings, but not with single embeddings, which should not overload participants’ memory capacity.

### Method

#### Participants.

Eighty-five native speakers of European Spanish participated in the experiment. They were recruited through social media and among students at the University of Oviedo. Four participants were excluded due to failures in data recording, chance performance in the operation verification part of the working memory task, and/or reporting a reading impairment. Additionally, one participant was excluded due to failure in responding within the response deadline on approximately 50% of the trials of the acceptability task. The remaining 80 participants had a mean age of 24 years (range: 17–40 years). Forty-nine self-identified as female and 31 as male. Nine were left-handed.

#### Materials.

Experimental materials comprised 24 items similar to those from Experiment 1, manipulated for grammaticality and number of embeddings. The design differed from Experiment 1 in two ways. First, single embeddings were included as conditions within the experimental items—rather than as separate items (Table 3). This allowed us to quantify the missing VP illusion as an interaction between grammaticality and number of embeddings. The interaction may provide a more accurate measure of the illusion because it takes into account differences in sentence length between grammatical and ungrammatical sentences. Second, we only tested clauses without inversion, because they yielded the strongest missing VP illusion in Experiment 1. We wanted to elicit the largest possible illusion to be able to probe for modulations due to the participants’ memory capacity.

**Table 3.**
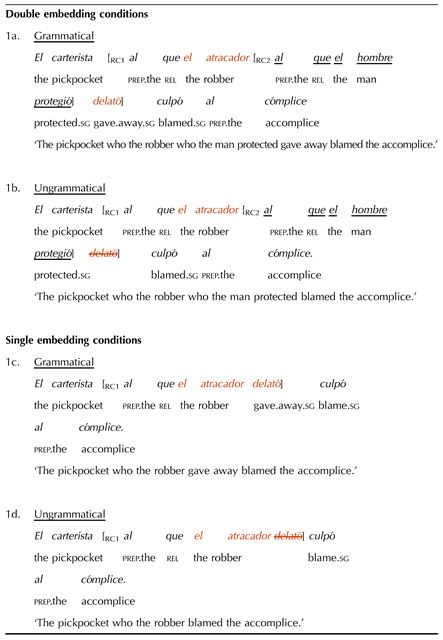
Sample item set in Experiment 2

*Note*. The critical verb is crossed-out in the ungrammatical conditions to represent its omission. The clause that was eliminated in the single embedding conditions is underlined.

#### Procedure.

The experiment was conducted in person on two laptop PCs. After completing a demographic questionnaire, participants performed a speeded acceptability judgment task similar to that of Experiment 1. Experimental sentences were intermixed with 48 fillers and 32 items from a separate experiment (not reported here).

After the acceptability task, participants performed an operation span task that measured their working memory capacity. This task involves recalling series of items while solving mathematical operations (Turner & Engle, 1989). The task was adapted from von der Malsburg (2015). Participants read equations like (2 + 7) × 5 = 45 aloud and indicated whether they were correctly solved by pressing the keys *F* and *J* (‘no’ and ‘yes’, respectively). After each equation, a consonant was shown on screen for 1000 ms. Each sequence of equation and consonant occurred between three and five times across trials. Afterwards, participants had to write the letters they remembered in their order of appearance. There were fifteen trials, presented in a randomized order. Before the memory task, there was a pretest in which participants were asked to verify whether fifteen equations were correctly solved. The pretest was presented as a practice and participants received feedback after each operation. However, the main purpose of this part (hidden from participants), was to measure the mean time each individual spent verifying an equation. This was to establish a personalized time limit for the following task (the mean response time + 2.5 standard deviations), such that fast equation solvers did not have extra time to rehearse the consonants before pressing the yes/no buttons. The operation span task took about 20–25 minutes. An entire experimental session lasted 35–45 minutes.

#### Analysis.

Following Conway et al. (2005), the operation span task was scored with the partial credit unit method: Credit was given when two or more consonants in a sequence were recalled in the correct order, irrespective of sequence length (e.g., a participant received a credit of 50% whether they recalled 1 out of 2 items or 2 out of 4 items). These by-participant working memory scores were used as predictors in the analysis of the acceptability task.

The analysis of the acceptability data followed Experiment 1. Responses with reaction times shorter than 100ms or longer than 2000ms were excluded: This affected 3.80% of the data (SD = 5.97%). The statistical analysis used the same priors and a maximal random effects structure. We used the same priors as in Experiment 1 because we did not know whether the results of Experiment 1 would replicate given the change in the procedure (web-based vs. in-person testing) and population (Prolific participants vs. university students). The main model included the factors grammaticality (−0.5 grammatical / 0.5 ungrammatical), number of embeddings (−0.5 single / 0.5 double) and working memory score (continuous and centered), as well as their interactions. As in Experiment 1, a missing VP illusion should be indexed by an interaction between grammaticality and the number of embeddings. Crucially, if working memory modulates the missing VP illusion, we expected a three-way interaction between working memory, grammaticality and the number of embeddings. A nested model was used to estimate the effect of the predictors in single and double embeddings separately.

### Results

**Figure 2.**
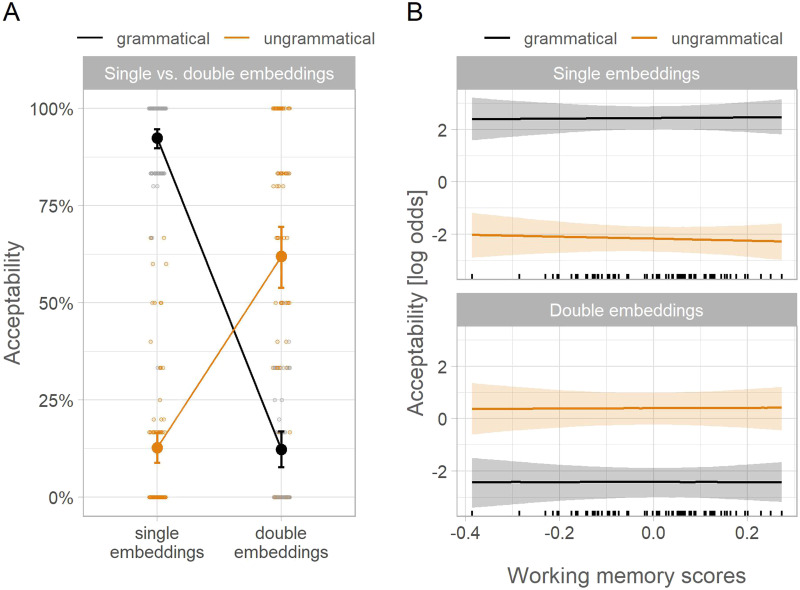
Acceptance rates in Experiment 2. *Note*. Panel A illustrates the missing VP effect in the empirical acceptance rates: With double embeddings, ungrammatical sentences were accepted more often than grammatical sentences, while the opposite was true of single embeddings. Larger points show empirical condition means across participants and error bars show 95% bootstrapped confidence intervals. Smaller points depict by-participant means. Panel B illustrates the model-estimated conditional effects of grammaticality and number of embeddings as a function of participants’ memory scores. Vertical black lines on the x-axis represent the number of participants at each point of the memory scale. Working memory capacity did not have a consistent effect on acceptability in either double or single embeddings, as shown by the constant effect of grammaticality across scores.

In the operation span task, participants’ mean accuracy verifying the operations was 92% (range across participants: 67–100%), and participants’ recall scores ranged between 27–93% (Mean = 65%; SD = 13%). In the acceptability task, mean accuracy in the filler items was 90% (range by participant: 73–100 %). The empirical acceptance rates of the experimental conditions are shown in Figure 2A. The model estimates are shown in Table 4 and they are reported back-transformed to percentages in the text.

**Table 4.** Model estimates in Experiment 2

	Estimate	95% CrI
Intercept (grand mean)	−0.45	[−0.77, −0.13]
Number of embeddings	−1.15	[−1.63, −0.67]
Grammaticality	−0.90	[−1.37, −0.43]
Working Memory	−0.03	[−1.46, 1.44]
Number of embeddings × Grammaticality	7.44	[6.49, 8.34]
Working Memory × Number of embeddings	0.14	[−1.53, 1.76]
Working memory: single embeddings	−0.15	[−1.58, 1.22]
Working memory: double embeddings	0.08	[−1.66, 1.83]
Working Memory × Grammaticality	−0.24	[−1.86, 1.43]
Working Memory × Number of embeddings × Grammaticality	0.51	[−1.33, 2.36]

*Note*. Columns show the estimated effect size (Estimate) with its 95% credible interval (CrI) in log-odds. For the factor Number of embeddings, a negative coefficient indicates lower acceptability in double embeddings. For Grammaticality, a negative coefficient indicates lower acceptability in ungrammatical sentences. For Working Memory, a negative coefficient indicates lower acceptability with increasing working memory.

#### The Missing VP Effect.

The comparison of sentences with single and double embeddings revealed a grammaticality × number of embeddings interaction: 95 [91, 97] % (Table 4). This interaction indicated opposite grammaticality effects for single vs. double embeddings. With single embeddings, ungrammatical sentences were accepted less often than grammatical sentences, consistent with a canonical grammaticality effect: −68 [−79, −57] %. This pattern was reversed in double embeddings: Ungrammatical sentences were accepted more often than grammatical sentences, consistent with a missing VP effect: 26 [16, 37] % (Figure 2A).

#### The Effect of Working Memory.

Working memory did not have a systematic effect on the missing VP illusion: The credible interval of the working memory × grammaticality × number of embeddings interaction spanned negative and positive values: 12 [−31, 51] % with a probability of a positive interaction of 0.71. Similarly, working memory scores did not modulate the difference between grammatical and ungrammatical conditions when single and double embeddings were analyzed separately: 16 [−26, 53] % and 6 [−28, 40] % (Figure 2B). The probability of a positive working memory × grammaticality interaction was 0.22 for single embeddings and 0.63 for double embeddings.

### Discussion

Experiment 2 successfully replicated the missing VP illusion in Spanish: Participants rated ungrammatical double embeddings as more acceptable than their grammatical counterparts, while the opposite pattern was observed with single embeddings. Meanwhile, working memory scores, as measured by an operation span task, did not have a consistent effect on participants’ susceptibility to the missing VP illusion. The absence of a consistent working memory modulation was further attested in both single and double embeddings.

Our failure to detect an effect of working memory could have different causes. A first possibility would be that memory limitations play no role in the illusion. We consider this unlikely, as it would be inconsistent with most existing accounts and it would mean shifting all explanatory power to alternative factors like language statistics (Christiansen & Chater, 1999; Christiansen & MacDonald, 2009; Engelmann & Vasishth, 2009; Frank & Ernst, 2019; Frank et al., 2016, 2021; Futrell et al., 2020; Futrell & Levy, 2017), prosody (Fodor et al., 2017; but note that our task involved silent reading) or repair processes by which thematic relations between all verbs and nouns are established (Huang & Phillips, 2021).

A more likely possibility is that working memory is indeed involved in the illusion, but that we failed to detect an effect due to either the nature of our materials or to limitations of the task that assessed working memory. With regard to our materials, the processing of double embeddings may have been too demanding to enable the detection of a working memory modulation. Specifically, Hofmeister et al. (2014) have reported that processing difficulty associated with low ratings is modulated by participants’ reading span scores only when a sentence’s difficulty is moderate (as in sentences with two non-overlapping dependencies) but not when it is very high (as in sentences in which one dependency crosses another). Our double embeddings may have been especially hard to process since all determiner phrases were third-person definite descriptions, which hamper processing in double embeddings as compared to pronouns (Gibson, 2000). This difficulty may have been further fostered by the speeded nature of the task.

However, if working memory did not modulate the missing VP illusion due to extreme processing difficulty, we should have observed a floor effect, with no variation in the size of the illusion across participants. This was not the case: Supplementary analyses showed that participants clearly differed in their susceptibility to the missing VP illusion (Appendix C). Thus, the fact that working memory scores failed to modulate the illusion is unlikely due to a lack of inter-individual variation in the size of the illusion, which would have been expected if the illusion was very large for all participants. We also think that the lack of a working memory modulation was unlikely due to insufficient variation in participants’ working memory scores, since these ranged descriptively between 27–93% with a standard deviation of 13% (Appendix C).

A final potential cause for our failure to detect a working memory modulation is that the operation span task may not have provided an adequate measure of the memory system relevant for linguistic processing. Even though the operation span task has been shown to predict language comprehension (Daneman & Merikle, 1996; Turner & Engle, 1989), most previous studies claiming that working memory predicts comprehension ability have instead used reading span tasks (Daneman & Carpenter, 1980). The reading span task differs from the operation span task in that participants read sentences instead of verifying equations. We did not use a reading span task due to concerns that it might measure language-internal working memory capacity or even reading comprehension ability (Baddeley et al., 1985; Caplan & Waters, 1999). Thus, our failure to find an effect may be due to our decision to use the operation span task as a more language-independent measure of working memory.

Alternatively, span tasks in general may not be adequate predictors of comprehension ability, since the ability they measure—e.g., maintenance capacity—does not play a significant role in comprehension under cue-based memory retrieval models (Lewis et al., 2006; McElree et al., 2003; Van Dyke et al., 2014). From this perspective, a task that measures participants’ ability to avoid interference may be more likely to predict the size of the illusion (see Friedman & Miyake, 2004 for a comparison between tasks).

## GENERAL DISCUSSION

Experiments 1 and 2 demonstrated that the missing VP illusion occurs in Spanish: Sentences with double embeddings were accepted more often when they lacked a verb phrase than when they contained all the verb phrases necessary to make the sentence grammatical. Since Spanish is a verb-initial language, our results align with previous findings suggesting that speakers of such languages experience the illusion. Notably, most previous findings supporting a link between verb-initiality and the illusion come from English (Christiansen & MacDonald, 2009; Frank & Ernst, 2019; Frazier, 1985; Gibson & Thomas, 1999), a language in which word order is mostly fixed and thus a reliable processing cue for determining relationships between constituents. Since word order is more variable in Spanish, our results suggest that the connection between verb-initiality and the illusion also holds for languages in which word order is a less reliable processing cue (Kail, 1989; MacWhinney, 2001; MacWhinney et al., 1984). This strengthens the claim that basic verb position may be a key property in determining a language’s sensitivity to the illusion.

While our finding of a missing VP illusion in Spanish aligns with previous results in English, the size of the missing VP illusion seems descriptively larger in Spanish than in previous English studies. In our experiments, ungrammatical sentences were accepted in the majority of cases and grammatical sentences were rejected most of the time. In contrast, both conditions obtained low ratings in previous studies on English, and the differences between them were sometimes not statistically significant (Christiansen & MacDonald, 2009; Frank & Ernst, 2019; Gibson & Thomas, 1999). These differences could be associated to contrasting properties of Spanish and English, but it is unclear which factors would predict them. A more likely explanation is that the contrast is related to differences between the experimental tasks. In the studies on English, sentences were presented without any time constraints, and participants rated their acceptability on a Likert scale. By contrast, the sentences in our experiments were presented word-by-word in a speeded paradigm and participants had a time limit to rate them as “acceptable” or “unacceptable”. Our speeded task may have increased the acceptability of ungrammatical sentences relative to the untimed tasks for two reasons. First, because it could place higher demands on working memory and increase the risk of a parsing failure—thus leading to more acceptance of ungrammatical sentences. Second, because the nature of binary choices could have had a polarizing effect, by increasing the acceptability of the ungrammatical conditions and lowering the acceptability of the grammatical conditions. Future studies using Likert ratings and/or reading-for-comprehension paradigms will be important to further diagnose the profile of the missing VP illusion in Spanish and its similarity with those of other languages.

### The Missing VP Illusion in Spanish is Modulated by Knowledge of Language Statistics

Our study also assessed the role of language statistics in the illusion. Language statistics accounts propose that the illusion occurs (at least in part) because removing a VP reduces the number of consecutive verbs, yielding a more frequent configuration (Frank & Ernst, 2019; Frank et al., 2016, 2021). To test this proposal, we manipulated subject-verb inversion in the most embedded clause of our double embeddings. We predicted that, if the infrequency of consecutive-verb patterns contributes to the illusion, inversion should reduce its size. The reason is that inversion reduces the number of consecutive verbs, yielding what a corpus search revealed to be a more frequent configuration in structurally similar sentences with single embeddings.

Our findings confirmed this prediction by showing a smaller missing VP illusion in inverted sentences. This is consistent with the view that consecutive-verb patterns and language statistics contribute to the illusion. If speakers’ knowledge about language statistics is the underlying source of the inversion effect in the missing VP illusion, our results would support the view that acceptability is influenced by linguistic experience (Bybee, 2006, 2007, 2010; Goldberg, 2007, 2013; Tomasello, 2009), which allows comprehenders to assess the likelihood that words belonging to specific categories cluster together in sentences (Bermel & Knittl, 2012; Bresnan, 2007; Dąbrowska, 2008; Flach, 2020; Francis, 2022; Lau et al., 2017; Sprouse et al., 2018). The impact of word cluster frequencies on judgments is likely due to the fact that comprehenders perceive sentences as linear strings. In a string, words establish relations of precedence and succession with each other that may be more or less frequent in language use. Based on their linguistic experience, comprehenders have knowledge of the probabilities of these word-to-word transitions, which can influence comprehenders’ acceptability judgments. Under classical generative approaches, this knowledge is a performance factor (in the sense of Chomsky, 1965) that falls outside the scope of grammar. The finding that it can still affect judgments helps explain some misalignments between acceptability and grammaticality observable in illusions, and we think that more research should be devoted to understanding such effects.

While we favor a language statistics explanation of the inversion effect, we acknowledge that it might be also explained under some types of memory-based accounts. For example, under Dependency Locality Theory (Gibson, 2000), the inverted conditions might have a lower integration cost and reduce memory load. Further work measuring sentence reading times is needed to arbitrate between statistics- and memory-based explanations of the effect of inversion in the missing VP illusion.

### No Evidence That Spanish Participants’ Memory Span Explains Variability in the Missing VP Illusion

Many accounts, including language statistics accounts, also claim that working memory limitations contribute to the illusion (Bader, 2016; Frank & Ernst, 2019; Frank et al., 2021; Futrell et al., 2020; Futrell & Levy, 2017; Gibson & Thomas, 1999; Häussler & Bader, 2015; Vasishth et al., 2010). To evaluate this claim, we assessed whether working memory scores—measured in an operation span task—modulated the size of the illusion. Our hypothesis was that high-capacity participants should be more resilient to the illusion than their lower capacity peers. However, we did not find any indication that working memory scores affected the missing VP illusion size. We considered several possible reasons for this. In our view, the most compelling one is that the operation span task could perhaps not have tapped into the relevant memory construct. It seems less likely that we failed to detect a relationship between the illusion and working memory because there was insufficient inter-individual variation in either the acceptability task or the memory task, since both the illusion size and the working memory scores varied across participants.

We also consider it unlikely that our findings reflect a lack of involvement of working memory in the illusion, as working memory plays an explanatory role in most accounts. In our view, a promising way to account for the illusion and its cross-linguistic variation is through models that invoke both memory representations and expectations derived from language statistics. One such model is lossy context surprisal, where the processing difficulty of a word depends on how unexpected it is given a lossy—i.e., incomplete and/or faulty—memory representation of the preceding context (Futrell et al., 2020). Crucially, comprehenders need to complete the lossy memory representations of the preceding context to make predictions about the upcoming words, and they do so based on their language statistics knowledge. If the preceding context has an infrequent structure, as with double embeddings, comprehenders are likely to reconstruct it inaccurately and make incorrect predictions. This can explain the missing VP illusion if, for example, a faulty memory representation of *The report that the doctor* is reconstructed as *The report by the doctor*, which requires one less verb. The finding that verbs are more likely to be omitted when the first noun is unlikely to embed a clause in Spanish, English and German production (Hahn et al., 2022) supports this possibility, and it suggests that examining variation in the statistical patterns associated to lexical items and grammatical constructions is a fruitful research pathway to explore the interaction between language statistics and memory constraints.

### Conclusion

Acceptability judgments are an essential tool to describe human grammars, which makes it important to understand discrepancies between acceptability and grammaticality. Our study explored one such case, the missing VP illusion in Spanish, a language in which the illusion had not been previously tested in comprehension. We demonstrated that the illusion exists in Spanish, which suggests that speakers of verb-initial languages are susceptible to it, even in languages in which word order is not a particularly reliable processing cue. We further found that the frequency of consecutive verb sequences modulated the strength of the illusion, while comprehenders’ memory spans did not. Our results underscore the importance of considering the role of language statistics—like comprehenders’ knowledge about the probability of verb clusters—when using acceptability measures as a proxy for grammaticality.

## ACKNOWLEDGMENTS

We thank Edward Gibson, Titus von der Malsburg and the reviewers of this paper for their useful feedback and constructive suggestions. This research was partially supported by a “Margarita Salas” grant (Ayudas para la recualificación del Sistema Universitario español, convocatoria complementaria la de 2021–2023) awarded to Claudia Pañeda by the Ministerio de Universidades (Spain) and funded by the European Union (NextGenerationEU).

## AUTHOR CONTRIBUTIONS

Claudia Pañeda: Conceptualization: Equal; Data curation: Lead; Formal analysis: Supporting; Investigation: Lead; Methodology: Equal; Software: Equal; Writing – original draft; Writing – review & editing. Sol Lago: Conceptualization: Equal; Formal analysis: Lead; Methodology: Equal; Software: Equal; Writing – review & editing.

## Notes

^1^ Note that other types of memory account attribute the missing VP illusion to similarity-based interference, rather than a capacity overload (Bader, 2016; Häussler & Bader, 2015). We do not provide a detailed explanation of the different types of memory accounts because our experiments cannot distinguish between them. For our purposes, the key point is that all accounts assign a central role to working memory capacity and thus predict that it should influence the missing VP illusion—regardless of whether the underlying cognitive mechanism is overload or interference.^2^ This does not mean that speakers of such languages do not experience the illusion at all. Indeed, the illusion appears in reading times in German in configurations where the embedded clause does not modify a clause-initial determiner phrase—as in (1)—, but a clause-medial determiner phrase instead (Häussler & Bader, 2015). This configuration also increases the acceptability of missing VP double embeddings, but crucially not enough to make them equally or more acceptable than their grammatical counterparts, in contrast with English (Bader, 2016; Häussler & Bader, 2015).^3^ Evidence from French, which is also a verb-initial language, is mixed: Gimenes et al. (2009) found the illusion in judgments but not in reading times, which followed the German/Dutch pattern.

## Supplementary Material


