# Supplementary material for: The Missing VP Illusion in Spanish: Assessing the Role of Language Statistics and Working Memory
Source: Open Mind (Camb). 2024 Feb 1;8:42–66. doi: 10.1162/opmi_a_00118 (PMC10898615; doi:10.1162/opmi_a_00118)
Supplement: Supplementary file 1 [file opmi-08-42-s001.docx]

# Appendix A. Information on the CORPES XXI search

The corpus search was conducted in September 2022, in version 0.94 of the CORPES XXI (published in July 2021). We looked for structures that contained at least one center-embedded relative clause with either a non-inverted subject-verb order (i-a) or an inverted verb-subject order (i-b). This could potentially return sentences with single embeddings as well as sentences with double embeddings, since the outer constituents could be themselves part of an embedded relative clause.

| (i) |  | a. | S_j_ [_RC_ *that*_j_ S V] V |
| --- | --- | --- | --- |
|  |  | b. | S_j_ [_RC_ *that*_j_ V S] V |

However, we could not directly search for sentences with these structures, because CORPES XXI is not syntactically annotated. Instead, it contains part of speech tags and lemmas. Because of this, we searched for part of speech sequences that were compatible with the structures of interest. Then, we manually assessed whether the retrieved sequences corresponded to those structures. In what follows, we describe these two steps in detail.

## STEP 1: Search of part of speech sequences

To find sentences with the structure (ii-a), we searched for the sequence under A in **Table A1**. To find sentences with the structure (ii-b), we searched for the sequence under B.

**Table A1**

Part of speech sequences that were searched in CORPES XXI to find each of the target structures

| **A.** |  |  |  |  |  |
| --- | --- | --- | --- | --- | --- |
| *Searched sequence:* | | | | | |
| relative pronoun (lemma: *que*) | | definite article | noun | verb | verb  (NOT simple infinitive, NOT complex infinitive, NOT simple gerund, NOT complex gerund, NOT past participle) |
| 4 | | 3 | 2 | 1 |  |
| \| *Target structure:* S_j_ [_RC_ *that*_j_ S V] V \| \| --- \| | | | | | |
| **B.** | | | | | |
| *Searched sequence:* | | | | | |
| relative pronoun  (lemma: *que*) | | verb | definite article | noun | verb  (NOT simple infinitive, NOT complex infinitive, NOT simple gerund, NOT complex gerund, NOT past participle) |
| 4 | | 3 | 2 | 1 |  |
| *Target structure:* S_j_ [_RC_ *that*_j_ V S] V | | | | | |

*Note.* The numbers indicate the position that words tagged with the label above them should occupy relative to the final verb (e.g., in B, 1 underneath “noun” indicates that the search should return sequences in which there was a noun at the first position to the left of the verb).

In each case, we searched for words tagged as verbs (the rightmost part of speech in each sequence) with four left collocates. For each collocate, we specified the part of speech (relative pronoun, definite article, noun or verb). In the case of relative pronouns, we additionally specified the lemma (“que”), to restrict the search to cases with the same relative pronoun as in our experimental materials. The position of the collocates in the sequence was specified by indicating its position in the sequence relative to the verb (indicated in the table below each collocate).

As **Table A1** shows, the search excluded cases in which the final verb was a simple infinitive, a complex infinitive, a simple gerund, a complex gerund or a past participle. This was meant to exclude most of the cases in which the two verbs did not belong to different clauses, as in *Phil la convence prometiendo llevarla a una exposición que la niña quería ver* (‘Phil convinces her by promising to take her to an exhibition that she wanted to see’).

Search A retrieved 250 cases and search B retrieved 2354 cases.

## STEP 2: Manual assessment of the retrieved sequences

We then assessed whether the retrieved sequences corresponded to the target structures. Specifically, we checked whether each sequence met the following criteria:

1. **The definite article + noun sequence conformed the subject of the relative clause**, as in *La conexión emocional que genera el contacto contribuye a que confiemos más en los demás* (‘The emotional connection that contact generates helps us to trust others more’). This excluded cases like *El chófer que conducía el vehículo ha sido detenido* (‘The chauffeur that drove the vehicle has been arrested’), where the sequence was the object rather than the subject.
2. **The first verb of the sequence was inflected and headed the relative clause**. This excluded cases like *Todos destacaban el precio incalculable que el manuscrito robado podría alcanzar en el mercado negro de antigüedades* (‘All stressed the incalculable price that the stolen manuscript could reach on the antiques black market’), where the first verb, *robado* (‘stolen’) is uninflected and does not head the relative clause.
3. **The relative clause had an antecedent**, as in *Los tipos de interés que marca la Fed comenzarán a subir a mediados de 2015* (‘The interest rates that the Fed sets will begin to rise in mid-2015’, *Los nueve presos a los que entrevistó la BBC han dado testimonios "consistentes" de torturas* (‘The nine prisoners who the BBC interviewed have given “consistent” testimonies of torture’) or *La zona en la que está el chalet es muy arbolada* (‘The area in which the villa is located is very wooded’). This excluded sentences with free relatives like *Lo que obtiene el espectador es una especie de gratificación inmediata* (‘What the viewer gets is a kind of immediate gratification’).
4. **The antecedent was the subject of the outer verb.** This excluded cases like *Quien controla los mensajes que recibe la masa controla el poder* (‘Whoever controls the messages that the masses receive controls power’), *Para un acercamiento a los municipios que integran el proyecto es necesario matizar que son aquellos que conforman la frontera administrativa con Portugal* (‘For an approach to the municipalities that make up the project, it is necessary to clarify that they are those that form the administrative border with Portugal’) or *Una de las explicaciones que proponen los investigadores es que cuando se amamanta a los niños, están expuestos a una amplia variedad de sabores a través de la leche materna* (‘One of the explanations that researchers propose is that when infants are breastfed, they are exposed to a wide variety of flavors through breast milk’). We additionally excluded cases with the main verb *ser* (‘to be’) in which it was not clear whether the antecedent was to be interpreted as a subject or as an attribute, e.g., *El punto de fuerza del que arranca la novela es un crimen terrible y la subsiguiente investigación* (‘The point from which the novel starts is a terrible crime and the subsequent investigation’).

We additionally excluded cases that did not correspond to the desired structure because they were erroneously coded, e.g., the *que* was a complementizer rather a relative pronoun or one of words tagged as verb was a homophonous word instancing a different part of speech.

After filtering the returned cases in this way, we were left with 32 sentences without inversion and 436 sentences with inversion, as indicated in the main text. All were single embeddings.

# Appendix B. Sentence materials used in Experiments 1 and 2

## Experiment 1

### Double center-embedded item sets

The crossed-out verb was present in the grammatical conditions and removed in the ungrammatical conditions. The underlined clause appeared in the form to the left of the slash in the non-inverted conditions and in the form to the right of the slash in the inverted conditions.

| (1) | *El carpintero* [_RC1_ *al que el electricista* [_RC2_ *al que los propietarios llamaron* / *al que llamaron los propietarios*] *~~ayudó~~*] *humilló al aprendiz.*  ‘The carpenter who the electrician who the owners called helped humiliated the apprentice.’ |
| --- | --- |
| (2) | *El escritor* [_RC1_ *al que el famoso* [_RC2_ *al que los internautas estigmatizaron* / *al que estigmatizaron los internautas*] *~~apoyó~~*] *descalificó al cantante.*  ‘The writer who the celebrity who the internet users stigmatized supported disqualified the singer.’ |
| (3) | *El sargento* [_RC1_ *al que el enemigo* [_RC2_ *al que los soldados capturaron* / *capturaron los soldados*] *~~hirió~~*] *interrogó al civil.*  ‘The sergeant who the enemy who the soldiers captured hurt interrogated the civilian.’ |
| (4) | *El delegado* [_RC1_ *al que el maestro* [_RC2_ *al que los alumnos desobedecieron* / *al que desobedecieron los alumnos*] *~~nombró~~*] *delató al cabecilla.*  ‘The student representative who the teacher who the pupils disobeyed nominated gave away the leader.’ |
| (5) | *El músico* [_RC1_ *al que el adolescente* [_RC2_ *al que los novios invitaron* / *al que invitaron los novios*] *~~zarandeó~~*] *responsabilizó al camarero.*  ‘The musician who the teenager who the bride and groom invited shook blamed the waiter.’ |
| (6) | *El saxofonista* [_RC1_ *al que el director* [_RC2_ *al que los músicos homenajearon* / *al que homenajearon los músicos*] *~~alabó~~*] *perjudicó al violinista.*  ‘The saxophonist who the director who the musicians honored praised harmed the violinist.’ |
| (7) | *El político* [_RC1_ *al que el portavoz* [_RC2_ *al que los reporteros interrumpieron* / *al que interrumpieron los reporteros*] *~~desmintió~~*] *despidió al asesor.*  ‘The politician who the spokesman who the reporters interrupted refuted fired the advisor.’ |
| (8) | *El hombre* [_RC1_ *al que el detective* [_RC2_ *al que los vecinos contrataron* / *al que contrataron los vecinos*] *~~siguió~~*] *estafó al anciano.*  ‘The man who the detective who the residents hired followed ripped the old man off.’ |
| (9) | *El fotógrafo* [_RC1_ *al que el actor* [_RC2_ *al que los comentaristas ofendieron* / *al que ofendieron los comentaristas*] *~~pegó~~*] *llamó al abogado.*  ‘The photographer who the actor who the commentators offended hit called the lawyer.’ |
| (10) | *El turista* [_RC1_ *al que el hombre* [_RC2_ *al que los agentes liberaron* / *al que liberaron los agentes*] *~~estafó~~*] *recurrió al cónsul.*  ‘The tourist who the man who the policemen freed ripped off turned to the consul.’ |
| (11) | *El funcionario* [_RC1_ *al que el responsable* [_RC2_ *al que los inspectores avisaron* / *al que avisaron los inspectores*] *~~destituyó~~*] *sobornó al administrativo.*  ‘The clerk who the person in charge who the inspectors notified dismissed from his post bribed the administrative officer.’ |
| (12) | *El galerista* [_RC1_ *al que el político* [_RC2_ *al que los manifestantes presionaron* / *al que presionaron los manifestantes*] *~~llamó~~*] *censuró al pintor.*  ‘The gallery owner who the politician who the demonstrators pressured called censured the painter.’ |
| (13) | *El pianista* [_RC1_ *al que el experto* [_RC2_ *al que los organizadores menospreciaron* / *al que menospreciaron los organizadores*] *~~recomendó~~*] *conmovió al rey.*  ‘The pianist who the expert who the organizers scorned recommended moved the king.’ |
| (14) | *El intermediario* [_RC1_ *al que el empresario* [_RC2_ *al que extorsionaron los mafiosos* / *al que extorsionaron los mafiosos*] *~~pagó~~*] *disparó al testigo.*  ‘The middleman who the businessman who the gangsters extorted payed shot the witness.’ |
| (15) | *El magistrado* [_RC1_ *al que el fiscal* [_RC2_ *al que desautorizaron los abogados* / *al que desautorizaron los abogados*] *~~irritó~~*] *indultó al infractor.*  ‘The magistrate who the prosecutor who the lawyers discredited irritated pardoned the offender.’ |
| (16) | *El secuestrador* [_RC1_ *al que el negociador* [_RC2_ *al que convocaron los investigadores* / *al que convocaron los investigadores*] *~~convenció~~*] *soltó al rehén.*  ‘The kidnapper who the negotiator who the investigators called convinced released the hostage.’ |
| (17) | *El intérprete* [_RC1_ *al que el parlamentario* [_RC2_ *al que aconsejaron los asesores* / *al que aconsejaron los asesores*] *~~eligió~~*] *insultó al embajador.*  ‘The interpreter who the member of parliament who the advisors advised chose insulted the ambassador.’ |
| (18) | *El carterista* [_RC1_ *al que el atracador* [_RC1_ *al que los hombres protegieron* / *al que protegieron los hombres*] *~~acusó~~*] *culpó al cómplice.*  ‘The pickpocket who the robber who the men protected accused blamed the accomplice.’ |
| (19) | *El estudiante* [_RC1_ *al que el director* [_RC2_ *al que informaron los profesores* / *al que informaron los profesores*] *~~infravaloró~~*] *sorprendió al examinador.*  ‘The student who the principal who the teachers informed underestimated surprised the examiner.’ |
| (20) | *El guardabosques* [_RC1_ *al que el campesino* [_RC2_ *al que los senderistas abordaron* / *al que abordaron los senderistas*] *~~avisó~~*] *curó al herido.*  ‘The forest ranger who the peasant who the hikers approached informed healed the injured person.’ |
| (21) | *El bailarín* [_RC1_ *al que el coreógrafo* [_RC2_ *al que los espectadores aplaudieron* / *al que aplaudieron los espectadores*] *~~discriminó~~*] *recibió al periodista.*  ‘The dancer who the choreographer who the members of the audience applauded discriminated received the journalist.’ |
| (22) | *El testigo* [_RC1_ *al que el agente* [_RC2_ *al que los investigadores seleccionaron* / *al que seleccionaron los investigadores*] *~~condicionó~~*] *confundió al culpable.*  ‘The witness who the agent who the investigators selected influenced confused the culprit.’ |
| (23) | *El chico* [_RC1_ *al que el señor* [_RC2_ *al que los timadores engañaron* / *al que engañaron los timadores*] *~~incriminó~~*] *mintió al juez.*  ‘The guy who the gentleman who the swindlers deceived incriminated lied to the judge.’ |
| (24) | *El policía* [_RC1_ *al que el comisario* [_RC2_ *al que visitaron los denunciantes* / *al que visitaron los denunciantes*] *~~criticó~~*] *atrapó al ladrón.*  ‘The policeman who the captain who the accusers visited criticized caught the thief.’ |

### Single center-embedded item sets

Items (25) to (30) have plural subjects in the embedded clause, while items (31) to (36) have singular subjects. In each group, the first three items do not have subject-verb inversion, while the last three do.

| (25) | *Los contrincantes* [_RC1_ *a los que los ganadores ~~derrotaron~~*] *ofendieron a los televidentes.*  ‘The opponents who the winners defeated offended the television viewers.’ |
| --- | --- |
| (26) | *Los políticos* [_RC1_ *a los que los reporteros ~~ridiculizaron~~*] *increparon a los diputados.*  ‘The politicians who the reporters ridiculed rebuked the representatives.’ |
| (27) | *Los hosteleros* [_RC1_ *a los que los comerciantes ~~apoyaron~~*] *criticaron a los ministros.*  ‘The hoteliers who the storekeepers supported criticized the ministers.’ |
| (28) | *Los cardiólogos* [_RC1_ *a los que ~~avalaron~~ los cirujanos*] *consolaron a los familiares.*  ‘The cardiologists who the surgeons backed up comforted the relatives.’ |
| (29) | *Los árbitros* [_RC1_ *a los que ~~abuchearon~~ los socios*] *sancionaron a los futbolistas.*  ‘The referees who the members booed banned the football players.’ |
| (30) | *Los héroes* [_RC1_ *a los que ~~ensalzaron~~ los poetas*] *aburrieron a los lectores.*  ‘The heros who the poets exalted bored the readers.’ |
| (31) | *El socorrista* [_RC1_ *al que el bañista ~~salpicó~~*] *riñó al niño.*  ‘The lifeguard who the bather splashed scoled the boy.’ |
| (32) | *El humorista* [_RC1_ *al que el guionista ~~aplaudió~~*] *humilló al actor.*  ‘The comedian who the scriptwriter applauded humiliated the actor.’ |
| (33) | *El candidato* [_RC1_ *al que el ciudadano ~~votó~~*] *encubrió al concejal.*  ‘The candidate who the citizen voted for covered up for the city councilor.’ |
| (34) | *El sindicalista* [_RC1_ *al que ~~recurrió~~ el obrero*] *persuadió al inversor.*  ‘The union member who the worker turned to persuaded the investor.’ |
| (35) | *El presentador* [_RC1_ *al que ~~alabó~~ el comentarista*] *entrevistó al psicólogo.*  ‘The presenter who the commentator praised interviewed the psychologist.’ |
| (36) | *El teleoperador* [_RC1_ *al que ~~exasperó~~ el informático*] *desafió al jefe.*  ‘The telesales worker who the computer expert exasperated challenged the boss.’ |

### Filler items

| (37) | *El organista tocó una canción y luego el obispo bendijo a los fieles.*  ‘The organist played a song and then the bishop blessed the faithful.’ |
| --- | --- |
| (38) | *El casero bajaría el precio del alquiler si los inquilinos no destrozaran los muebles.*  ‘The landlord would lower the rent if the tenants did not destroy the furniture.’ |
| (39) | *La esquiadora se horrorizó al ver que un ciervo corría por la pista.*  ‘The skier was horrified to see a deer running down the slope.’ |
| (40) | *El apicultor fue al hospital para que le curaran unas picaduras de abeja.*  ‘The beekeeper went to the hospital to be treated for bee stings.’ |
| (41) | *Las chicas se quejaron de la tardanza del repartidor y este se disculpó.*  ‘The girls complained about the delivery man's tardiness and he apologized.’ |
| (42) | *Los espías acecharon al diplomático pese a que sus jefes les habían ordenado esperar.*  ‘The spies stalked the diplomat even though their bosses had ordered them to wait.’ |
| (43) | *Los reyes habrían anunciado la noticia si sus asesores se lo hubieran aconsejado.*  ‘The king and the queen would have announced the news if their advisors had advised them to do so.’ |
| (44) | *La cuidadora reprendió a la niña porque había empujado a su hermana.*  ‘The caregiver reprimanded the girl because she had pushed her sister.’ |
| (45) | *El librero convenció a la novelista para que diera una charla sobre su nueva obra.*  ‘The bookseller convinced the novelist to give a talk about her new work.’ |
| (46) | *El farmacéutico venderá ese medicamento a los clientes cuando la agencia lo apruebe.*  ‘The pharmacist will sell that drug to customers when approved by the agency.’ |
| (47) | *Los bomberos rescataron al gato y evitaron que se asfixiara en el incendio.*  ‘The firefighters rescued the cat and prevented it from suffocating in the fire.’ |
| (48) | *El rector comunicó a su gabinete que quería becar a estudiantes con resultados excelentes.*  ‘The president informed his cabinet that he wanted to award scholarships to students with excellent results.’ |
| (49) | *Las gimnastas desayunaron con la entrenadora después de hacer yoga.*  ‘The gymnasts had breakfast with the coach after yoga.’ |
| (50) | *La soprano no canceló su actuación aunque se lo había aconsejado el laringólogo.*  ‘The soprano did not cancel her performance even though she had been advised to do so by her laryngologist.’ |
| (51) | *El recluta dejó al coronel muy impresionado con lo que había aprendido.*  ‘The recruit left the colonel very impressed with what he had learned.’ |
| (52) | *El marinero se quedó anonadado cuando el capitán anunció el cambio de rumbo.*  ‘The sailor was stunned when the captain announced the change of course.’ |
| (53) | *Los forenses examinaron el cadáver a pesar de que la causa de la muerte era evidente.*  ‘Forensic examiners examined the body even though the cause of death was obvious.’ |
| (54) | *El camionero adelantó al motorista sin tener en cuenta que estaba prohibido*.  ‘The truck driver overtook the motorcyclist without taking into account that it was forbidden.’ |
| (55) | *La historiadora constató que el escultor no era muy conocido en los círculos académicos.*  ‘The historian noted that the sculptor was not well known in academic circles.’ |
| (56) | *El jardinero regó las flores del jardín porque el calor las había dejado mustias*.  ‘The gardener watered the flowers in the garden because the heat had made them wilted.’ |
| (57) | *El cocinero le dijo al pinche que necesitaba que los tomates estuvieran maduros.*  ‘The cook told the kitchen assistant that he needed the tomatoes to be ripe.’ |
| (58) | *Los vigilantes amonestaron a los presos porque estos se habían peleado en el comedor*.  ‘The guards reprimanded the prisoners because they had fighted in the dining room.’ |
| (59) | *El cartero llamó a voces al viandante porque tenía un paquete para él.*  ‘The letter carrier called out to the passerby because he had a package for him.’ |
| (60) | *Los políticos preguntaron a los epidemiólogos cuál era la magnitud del problema*.  ‘Politicians asked epidemiologists about the magnitude of the problem.’ |
| (61) | *Los arqueólogos excavaron toda la noche porque esperaban encontrar el sarcófago del faraón.*  ‘The archaeologists excavated all night because they hoped to find the sarcophagus of the pharaoh.’ |
| (62) | *El maquinista detuvo el tren porque creyó ver un animal en la vía.*  ‘The engineer stopped the train because he thought he saw an animal on the track.’ |
| (63) | *El millonario tenía tanto dinero que decidió pagarse un viaje al espacio exterior.*  ‘The millionaire had so much money that he decided to pay for a trip to outer space.’ |
| (64) | *El cerrajero cambió la cerradura después de que los ladrones se colaran en la casa.*  ‘The locksmith changed the lock after the burglars broke into the house.’ |
| (65) | *El aristócrata cedió sus terrenos para que el ayuntamiento pudiera construir la carretera.*  ‘The aristocrat ceded his land so that the municipality could build the road.’ |
| (66) | *El albañil se cayó del tejado al oír el grito de su compañero.*  ‘The bricklayer fell off the roof at the sound of his partner's shout.’ |
| (67) | *La costurera se pinchó el dedo porque se olvidó de ponerse el dedal.*  ‘The seamstress pricked her finger because she forgot to put on her thimble.’ |
| (68) | *La joven acarició a su perro mientras la veterinaria le curaba la pata.*  ‘The young woman petted her dog while the veterinarian treated her paw.’ |
| (69) | *La madre acunó al bebé para que dejara de llorar y se durmiera.*  ‘The mother rocked the baby so that he would stop crying and go to sleep.’ |
| (70) | *Los programadores ganaron un premio por haber creado un videojuego tan exitoso.*  ‘The programmers won an award for creating such a successful video game.’ |
| (71) | *El sastre confeccionó un traje para que el presidente pudiera asistir a la gala.*  ‘The tailor made a suit for the president to attend the gala.’ |
| (72) | *El astronauta se quitó el casco a pesar de que sin él no podía respirar.*  ‘The astronaut took off his helmet even though he could not breathe without it.’ |
| (73) | **La peluquera despachó a la señora antes de que las limpiadoras fregara el suelo.*  ‘The hairdresser sent the lady away before the cleaners mopped the floor.’ |
| (74) | **El vendedor llamó al proveedor porque la madrina no querían el vestido.*  ‘The seller called the supplier because the godmother did not want the dress.’ |
| (75) | **El masajista pidieron ayuda a la osteópata para aliviar la contractura de la paciente.*  ‘The massage therapist asked the osteopath for help to relieve the patient's contracture.’ |
| (76) | **La madre siguieron la receta de la abuela a pesar de que no era el cumpleaños del niño.*  ‘The mother followed the grandmother's recipe even though it was not the child's birthday.’ |
| (77) | **El doctor auscultó al enfermo después de que la enfermera le pusieran la inyección*.  ‘The doctor auscultated the patient after the nurse gave him the injection.’ |
| (78) | **Las escritoras llevó a sus hijas a la conferencia para que siguieran el ejemplo de las científicas.*  ‘The writers brought their daughters to the conference to follow the example of the scientists.’ |
| (79) | **El piloto contactó con el controlador cuando la embarazada se pusieron de parto*.  ‘The pilot contacted the controller when the pregnant woman went into labor.’ |
| (80) | **Los domadores fustigó al león pese a las críticas de los asistentes*.  ‘The tamers whipped the lion despite criticism from the audience.’ |
| (81) | **Los recepcionistas evacuaron a los huéspedes porque los arquitectos vio grietas en el hotel.*  ‘The receptionists evacuated the guests because the architects saw cracks in the hotel.’ |
| (82) | **La cajera se disculpó ante el cliente porque ayer le venda unos yogures caducados*.  ‘The cashier apologized to the customer for selling him expired yogurt yesterday.’ |
| (83) | **El cura instó a los feligreses a que asistirán a la iglesia en Pascua*.  ‘The priest urged parishioners to attend church on Easter.’ |
| (84) | **La florista vendió a la chica un ramo de flores para que regaló a su tía*.  ‘The florist sold the girl a bouquet of flowers to give to her aunt.’ |
| (85) | **Los mayores reclamaron a la administración que les vacunarían cuanto antes*.  ‘The elderly demanded that the administration vaccinated them as soon as possible.’ |
| (86) | **Los violentos hirieron al joyero y por eso pasen a disposición judicial*.  ‘The violent men injured the jeweler and for this reason they were taken to court.’ |
| (87) | **El dentista atendió al niño mientras el padre esperará fuera de la consulta*.  ‘The dentist saw the child while the father waited outside the office.’ |
| (88) | **La anciana se sometió a la operación de cataratas pese a que todavía vea bastante bien.*  ‘The elderly woman underwent cataract surgery even though she still saw quite well.’ |
| (89) | **La traductora devolverá el libro cuando encontraría los datos que necesita*.  ‘The translator will return the book when she finds the data she needs.’ |
| (90) | **El filósofo publicó un artículo donde reflexione sobre las prisas de la sociedad moderna.*  ‘The philosopher published an article reflecting on the rush of modern society.’ |
| (91) | **El barbero propuso al chico un estilo que estaba pasada de moda*.  ‘The barber suggested the boy a style that was out of fashion.’ |
| (92) | **Los ancianos advirtieron a los legisladores de que sus pensiones eran muy bajos*.  ‘The seniors warned legislators that their pensions were too low.’ |
| (93) | **La azafata llevó agua a las pasajeras que se habían sentido indispuestos*.  ‘The flight attendant brought water to the passengers who had been feeling unwell.’ |
| (94) | **El famoso animó a sus seguidores a que se dejaran el pelo larga para parecerse a él.*  ‘The celebrity encouraged his followers to grow their hair long to look like him.’ |
| (95) | **La joven tenista fue ovacionado cuando derrotó a la ganadora del año pasado.*  ‘The young tennis player received a standing ovation when she defeated last year's winner.’ |
| (96) | **El psicólogo encontró al jubilado más cansado y deprimida que en la última revisión.*  ‘The psychologist found the retiree more tired and depressed than at the last checkup.’ |
| (97) | **La mujer se puso muy contento cuando aceptaron a su hija en un máster muy selectivo.*  ‘The woman was overjoyed when her daughter was accepted into a highly selective master's program.’ |
| (98) | **La princesa se quedó atónito cuando le dijeron que tenía que renunciar a sus títulos.*  ‘The princess was stunned when she was told she had to give up her titles.’ |
| (99) | **La alumna no pudo terminar su trabajo y por ello parecía angustiado.*  ‘The student was unable to finish her work and therefore seemed distressed.’ |
| (100) | **Los ganaderos pidieron ayudas económicas porque no poder conservar sus explotaciones.*  ‘The farmers asked for financial assistance because they were unable to maintain their farms.’ |
| (101) | **Los pescadores salir a faenar a pesar de que los meteorólogos habían desaconsejado la navegación.*  ‘The fishermen went out to fish even though the meteorologists had advised against sailing.’ |
| (102) | **Los mineros continuaron su actividad aunque haber alerta por desprendimiento*.  ‘The miners continued their activity even though there was a landslide alert.’ |
| (103) | **Los huelguistas volverían al trabajo si la patronal aceptar algunas de sus condiciones.*  ‘The strikers would return to work if the employers accepted some of their conditions.’ |
| (104) | **La maquilladora arreglar a la actriz como ella le pidió que lo hiciera*.  ‘The make-up artist fixed the actress as she asked her to do.’ |
| (105) | **El oficial esposó al sospechoso que huir por la ventana.*  ‘The officer handcuffed the suspect who fled out the window.’ |
| (106) | **La pitonisa engatusó a la señora pese a que su hija la haber prevenido.*  ‘The fortune-teller tricked the lady even though her daughter had warned her.’ |
| (107) | **El conductor atropellar al corredor porque estaba mirando el móvil.*  ‘The driver hit the jogger because he was looking at his cell phone.’ |
| (108) | **La senadora traicionó a su compañera pese a que haber prometido apoyar su investidura.*  ‘The senator betrayed her colleague despite having promised to support her investiture.’ |

## Experiment 2

### Experimental items

The crossed-out verb was present in the grammatical conditions and removed in the ungrammatical conditions. The underlined clause was present in the double embedding conditions and removed in the single embedding conditions.

| (1) | *El carpintero [_RC1_ al que el electricista [_RC2_ al que el fontanero llamó] ~~golpeó~~] supervisó al aprendiz.*  ‘The carpenter who the electrician who the plumber called hit supervised the apprentice.’ |
| --- | --- |
| (2) | *La madre [_RC1_ a la que la hija [_RC2_ a la que la hermana asustó] ~~encontró~~] saludó a la abuela.*  ‘The mother who the daughter who the sister scared found greeted the grandmother.’ |
| (3) | *El sargento [_RC1_ al que el teniente [_RC2_ al que el coronel buscó] ~~hirió~~] interrogó al civil.*  ‘The sergeant who the lieutenant who the colonel looked for injured questioned the civilian.’ |
| (4) | *El profesor [_RC1_ al que el vendedor [_RC2_ al que el empresario contrató] ~~confundió~~]*  *molestó al inversor.*  ‘The teacher who the salesman who the businessman hired confused bothered the investor.’ |
| (5) | *El pintor [_RC1_ al que el músico [_RC2_ al que la niña abrazó] ~~alojó~~] encontró al fotógrafo.*  ‘The painter who the musician who the little girl hugged had stay found the photographer.’ |
| (6) | *El saxofonista [_RC1_ al que el trompetista [_RC2_ al que el director visitó] ~~distrajo~~] felicitó al violinista.*  ‘The saxophonist who the trumpetist who the director visited distracted congratulated the violinist.’ |
| (7) | *El farmacéutico [_RC1_ al que el óptico [_RC2_ al que el cliente saludó] ~~preocupó~~] interrogó al empleado.*  ‘The pharmacist who the optician who the client greeted worried interrogated the employee.’ |
| (8) | *El limpiador [_RC1_ al que el conserje [_RC2_ al que el médico reconoció] ~~golpeó~~] sorprendió al paciente.*  ‘The cleaner who the janitor who the doctor recognized hit surprised the patient.’ |
| (9) | *El bailarín [_RC1_ al que el cantante [_RC2_ al que el espectador elogió] ~~acompañó~~] saludó al portero.*  ‘The dancer who the singer who the member of the audience praised accompanied greeted the doorman.’ |
| (10) | *El artista [_RC1_ al que el deportista [_RC2_ al que el guarda reprendió] ~~molestó~~] ayudó al principiante.*  ‘The artist who the sportsman who the guard scolded bothered helped the novice.’ |
| (11) | *El funcionario [_RC1_ al que el secretario [_RC2_ al que el administrador olvidó] ~~ignoró~~] ayudó al viandante.*  ‘The civil servant who the secretary who the administrator forgot ignored helped the pedestrian.’ |
| (12) | *El hijo [_RC1_ al que el padre [_RC2_ al que el profesor recordó] ~~aburrió~~] visitó al abuelo.*  ‘The son who the father who the teacher remembered bored visited the grandfather.’ |
| (13) | *El compositor [_RC1_ al que el corista [_RC2_ al que el obrero ignoró] ~~pegó~~] traicionó al músico.*  ‘The composer who the chorister who the worker ignored hit betrayed the musician.’ |
| (14) | *El abogado [_RC1_ al que el fiscal [_RC2_ al que el espía miró] ~~sorprendió~~] convenció al juez.*  ‘The lawyer who the district attorney who the spy looked at surprised convinced the judge.’ |
| (15) | *El primo [_RC1_ al que el hermano [_RC2_ al que el campesino describió] ~~complació~~] perdonó al tío.*  ‘The cousin who the brother who the peasant described pleased forgave the uncle.’ |
| (16) | *El pintor [_RC1_ al que el músico [_RC2_ al que el amigo abrazó] ~~enfadó~~] elogió al poeta.*  ‘The painter who the musician who the friend hugged angered praised the poet.’ |
| (17) | *El mago [_RC1_ al que el acróbata [_RC2_ al que el espectador describió] ~~enfadó~~] visitó al payaso.*  ‘The magician who the acrobat who the member of the audience described angered visited the clown.’ |
| (18) | *El carterista [_RC1_ al que el atracador [_RC2_ al que el hombre protegió] ~~delató~~] culpó al cómplice.*  ‘The pickpocket who the robber who the man protected gave away blamed the accomplice.’ |
| (19) | *El banquero [_RC1_ al que el prestamista [_RC2_ al que el cliente acompañó] ~~enfureció~~] robó al casero.*  ‘The banker who the moneylender who the client accompanied infuriated robbed the landlord.’ |
| (20) | *El vecino [_RC1_ al que el senderista [_RC2_ al que el guardabosques buscó] ~~importunó~~] curó al herido.*  ‘The neighbor who the hiker who the forest ranger looked for bothered cured the injured man.’ |
| (21) | *El futbolista [_RC1_ al que el entrenador [_RC2_ al que el seleccionador invitó] ~~insultó~~] recibió al periodista.*  ‘The football player who the trainer who the coach invited insulted received the journalist.’ |
| (22) | *El estudiante [_RC1_ al que el profesor [_RC2_ al que el director felicitó] ~~despertó~~] pisó al conserje.*  ‘The student who the teacher who the director congratulated woke stepped on the janitor.’ |
| (23) | *El repostero [_RC1_ al que el panadero [_RC2_ al que el cocinero avisó] ~~mintió~~] echó al camarero.*  ‘The pastry cook who the baker who the cook notified lied fired the waiter.’ |
| (24) | *El policía [_RC1_ al que el comisario [_RC2_ al que el criminal abofeteó] ~~denunció~~] cacheó al recluso.*  ‘The policeman who the superintendent who the criminal slapped reported searched the inmate.’ |

### Filler items

| (33) | **Me pregunto que vendrás a verme pronto.*  ‘I wonder that you will come to see me soon.’ |
| --- | --- |
| (34) | **No sé que tendrás tiempo para todo.*  ‘I don't know that you will have time for everything.’ |
| (35) | **Me gustaría saber que habrá o no habrá examen.*  ‘I would like to know that there will be an exam or not.’ |
| (36) | **¿Dudas sobre que comer un plátano o una manzana?*  ‘Are you doubting that to eat a banana or an apple?’ |
| (37) | **Le interrogó sobre que tenía contacto con el ladrón.*  ‘He interrogated him about that he had contact with the robber.’ |
| (38) | **¿No sabes que es mejor el café o el té?*  ‘You don't know that it is better coffee or tea?’ |
| (39) | **¿No te enteraste de que hace frío o calor en Francia?*  ‘Didn't you find out that it is cold or hot in France?’ |
| (40) | **¿No puedes decidir que alquilar o comprar un piso?*  ‘Can't you decide that rent or buy a flat?’ |
| (41) | **No habéis dado ningunos discurso en la convención del partido.*  ‘You haven't given any discourse in the party's convention.’ |
| (42) | **No hemos pedido ninguna becas para financiar el doctorado.*  ‘We haven't asked for any scholarships to fund the PhD.’ |
| (43) | **¿Habéis presentado algún denuncia por el robo del lunes?*  ‘Have you made any complaint about Monday's robbery?’ |
| (44) | **No hemos firmado ninguna contrato para alquilar un coche.*  ‘We haven't signed any contract to rent a car.’ |
| (45) | **¿Habéis solicitado alguna permiso para reparar el tejado?*  ‘Have you asked for any permission to repair the roof?’ |
| (46) | **¿Habéis dado algún justificación para vuestro comportamiento?*  ‘Have you given any excuse for your behavior?’ |
| (47) | **¿Habéis recibido algún presupuestos para la obra?*  ‘Have you received any cost estimates for the construction?’ |
| (48) | **¿Habéis planteado algunas queja por la subida de tasas?*  ‘Have you filed any complaint about the fee raise?’ |
| (49) | **Te han mandado que lavas la camiseta a mano.*  ‘They have commanded you that you hand wash the t-shirt.’ |
| (50) | **Me han abrazado para que me siento mejor.*  ‘They have hugged me such that I feel better.’ |
| (51) | **¿Qué te han aconsejado que haces cuanto antes?*  ‘What have they advised you that you do as soon as possible?’ |
| (52) | **Te han convencido para que asistes a la fiesta.*  ‘They have convinced you to attend the party.’ |
| (53) | **Te han recomendado que te defiendes con vigor.*  ‘They have recommended that you defend yourself vigorously.’ |
| (54) | **¿Qué me has indicado que explico otra vez?*  ‘What have you told me to explain again?’ |
| (55) | **¿Qué me has sugerido que preparo para cenar?*  ‘What have you suggested that I prepare for dinner?’ |
| (56) | **¿A quién has rogado que se cuida durante el viaje?*  ‘Who have you asked to take care of herself during the trip?’ |
| (57) | **El policía le a Ana ordenó que condujese despacio.*  ‘The policeman commanded Ana to drive slowly.’ |
| (58) | **La profesora me a mí exigió que repitiese los deberes.*  ‘The teacher demanded that I repeat my homework.’ |
| (59) | **¿Quién te a ti advirtió de que hicieses ejercicio?*  ‘Who advised you to exercise?’ |
| (60) | **El niño me a mí prometió que se portaría mejor.*  ‘The boy promised me that he would behave better.’ |
| (61) | **¿Quién te a ti aseguró que no habría despidos?*  ‘Who assured you that there would be no dismissals?’ |
| (62) | **¿Quién le a Marta contó que estábamos en el extranjero?*  ‘Who told Marta that we were abroad?’ |
| (63) | **¿Quién le a Luis juró que no había nada que temer?*  ‘Who swore to Luis that there would be nothing to be afraid of?’ |
| (64) | **¿Quién te a ti engañó para que cayeras en la trampa?*  ‘Who deceived you to fall in the trap?’ |
| (65) | *Me pregunto si conseguiré un buen coche de segunda mano.*  ‘I wonder whether I will get a good second-hand car.’ |
| (66) | *¿No sabes si podrás asistir a la reunión con la jefa?*  ‘Don't you know whether you'll be able to attend the meeting with the boss?’ |
| (67) | *Me gustaría saber si disponen de una hoja de reclamaciones.*  ‘I would like to know whether a complaint form is available.’ |
| (68) | *Dudo sobre si llevar un abrigo o solo una chaqueta.*  ‘I am doubting whether I should take a coat or only a jacket.’ |
| (69) | *No he presentado ninguna ponencia en el congreso de historia antigua.*  ‘I haven't given any talk at the ancient history conference.’ |
| (70) | *No has expresado ninguna opinión acerca del cambio de gobierno.*  ‘You haven't expressed any opinion about the government change.’ |
| (71) | *No he recibido ningún reconocimiento a pesar de la repercusión de mi trabajo.*  ‘I haven't received any acknowledgment despite the implications of my work.’ |
| (72) | *No has suspendido ningún examen a pesar de no haber estudiado.*  ‘You haven't failed at any exam even though you didn’t study.’ |
| (73) | *Me han llamado para que vaya a una entrevista.*  ‘I have been called to an interview.’ |
| (74) | *¿Te han deseado que tengas suerte en la competición?*  ‘Have they wished you luck for the competition?’ |
| (75) | *Me han animado para que me presente al concurso.*  ‘I have been encouraged to participate in the competition.’ |
| (76) | *Te han insistido para que nos hagas una visita.*  ‘You have been insisted upon to pay us a visit.’ |
| (77) | *El conserje le explicó a Rubén que el museo estaba cerrado.*  ‘The janitor explained to Rubén that the museum was closed.’ |
| (78) | *La funcionaria me reiteró a mí que el plazo había finalizado.*  ‘The clerk told me again that the delivery period had finished.’ |
| (79) | *El secretario te recordó a ti que el miércoles era festivo.*  ‘The secretary reminded you that there was a bank holiday on Wednesday.’ |
| (80) | *El banquero le repitió a Elisa que ya abrían cuentas sin comisiones.*  ‘The banker told Elisa again that the bank was already opening comission-free accounts.’ |

# Appendix C. Assessment of between-participant variability in the working memory scores and the missing VP illusion

Two steps were taken to ensure that the lack of a working memory modulation in the analysis of Experiment 2 was not due to lack of variability in either the predictor of interest (by-participant working memory scores) or the dependent variable (acceptance rates). With regard to working memory scores, a descriptive summary suggested that participants differed from one another in their performance of the span task: the by-participant scores ranged between 27–93%, with a mean of 66% and a standard deviation of 13% (**Figure C1**).

**Figure C1**

*Density plot of the distribution of working memory scores across participants in Experiment 2*


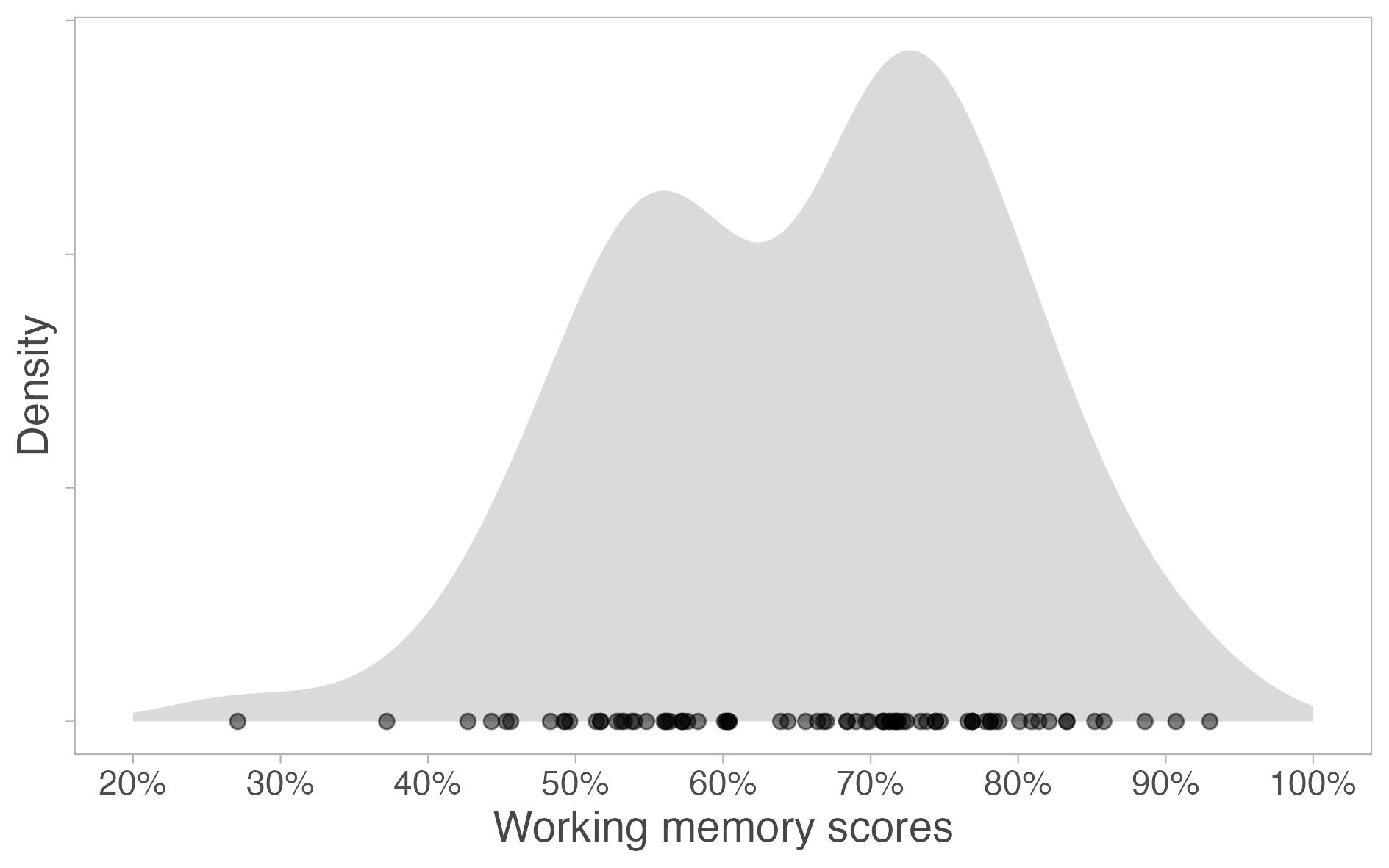


*Note.* Each dot represents a participant.

With regard to variability in acceptance rates, we conducted a supplementary analysis to examine whether participants differed in their susceptibility to the missing VP illusion. For this purpose, the missing VP illusion was operationalized as a grammaticality × number of embeddings interaction, which quantified the increased acceptability of ungrammatical vs. grammatical double embeddings, in the presence of an opposite effect in single embeddings (a canonical grammaticality effect).

To address the presence of between-participant variability in the missing VP effect, two models were compared in terms of their goodness-of-fit. The first model—used in the main article—included a grammaticality × number of embeddings by-participant random slope, which formalized the claim that participants differed in the size of the grammaticality × number of embeddings interaction (a *variable missing VP model*). This model was compared with a model that was identical except for lacking a grammaticality × number of embeddings by-participant random slope (a *baseline model*). The comparison assessed whether the variable missing VP model, which incorporated between-participant variability, provided a better fit to the data, despite its increased number of parameters.

We compared the two models in terms of their expected log pointwise density (ELPD), which is a measure of a model’s predictive accuracy if applied to a new dataset. ELPDs are computed by estimating how well each data point is predicted from all others, i.e., if the datapoint was removed. This procedure is called leave-one-out cross-validation (LOO; Vehtari et al. 2017). A lower ELPD indicates lower predictive accuracy.

In Experiment 1, the variable missing VP model provided a better fit to the data. The ELPD of the baseline model was smaller (ELPD difference = −27.0) with a difference greater than 6.1 standard errors relative to the variable model (a difference greater than 2 SEs suggests that one model is better than the other (Bürkner 2017; Vasishth et al. 2018). Similarly, in Experiment 2, the variable missing VP model also provided a better fit to the data, with a smaller ELPD in the baseline model (ELPD difference = −14.0; SE = 4.9).

In sum, model comparisons in Experiments 1 and 2 supported the existence of between-participant variability in the size of the missing VP illusion. This suggests that participants indeed varied in their susceptibility to the illusion, and thus that the absence of an effect of their working memory scores is unlikely to have resulted from a lack of underlying between-participant variability to be explained. **Figure C2** illustrates the amount of between-participant variability by showing the estimated size of the missing VP effect per participant.

**Figure C2**

*Model estimates of the grammaticality × number of embeddings interaction (x-axis) for each participant (y-axis) in Experiment 2*


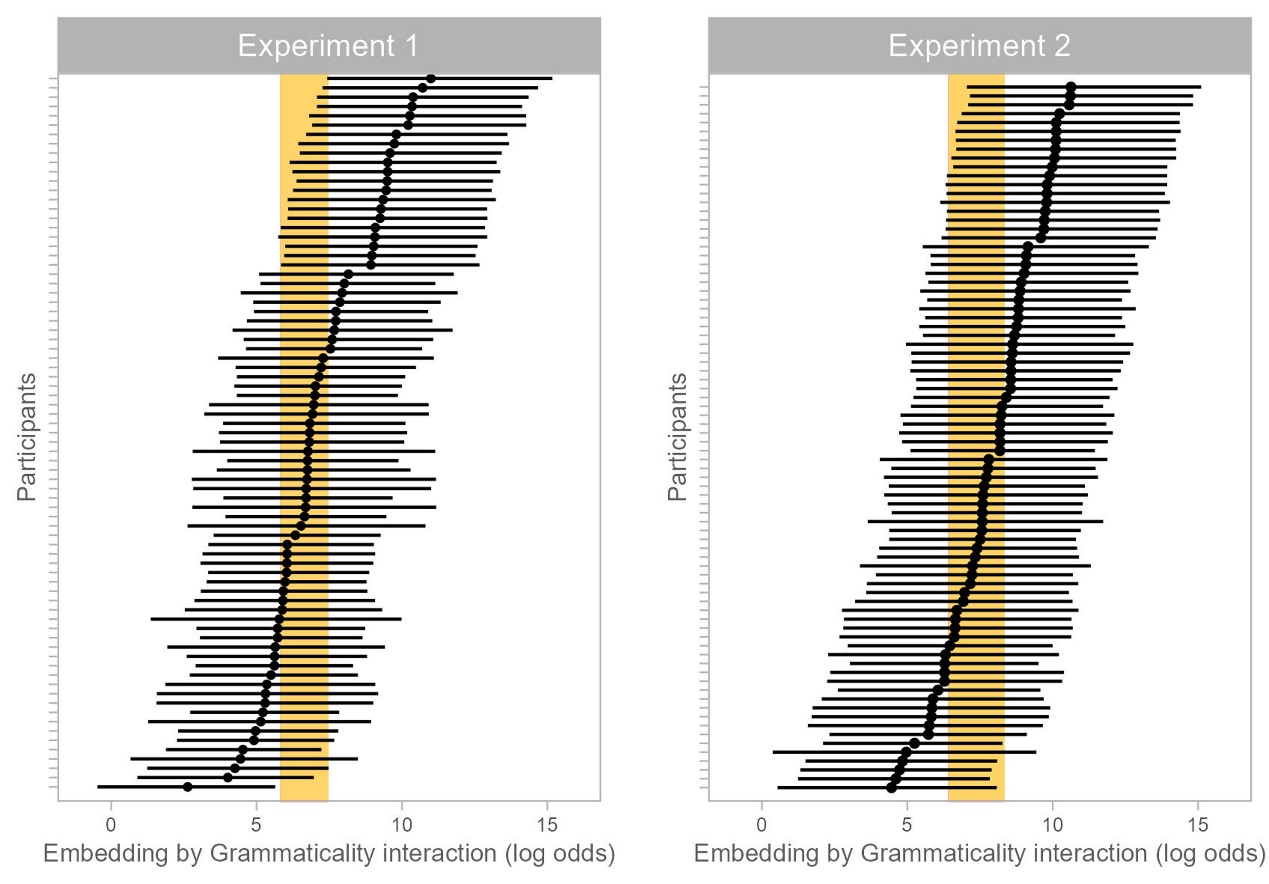


*Note.* The interaction is taken as an indication of the missing VP illusion, with smaller interactions representing smaller illusions. Points depict by-participant means and bars depict 95% credible intervals. Note that sizes are expressed in log odds because the effects are taken from the statistical model. The shaded orange area represents the mean and 95% credible interval of the grammaticality × number of embeddings interaction in the group data. The figure shows that the size of the missing VP illusion varied substantially across participants, with some participants showing larger and some showing smaller missing VP effects.

# References for Appendix

Bürkner, P.-C. ( 2017). brms: An R package for Bayesian multilevel models using Stan. *Journal of Statistical Software, 80*(1), 1–28. DOI: https://doi.org/10.18637/jss.v080.i01

Vasishth, S., Nicenboim, B., Beckman, M. E.; Li, F., & Kong, E. J. (2018). Bayesian data analysis in the phonetic sciences: A tutorial introduction. *Journal of Phonetics, 71*, 147–161. DOI: https://doi.org/10.1016/J.WOCN.2018.07.008

Vehtari, A., Gelman, A., & Gabry, J. (2017). Practical Bayesian model evaluation using leave-one-out cross-validation and WAIC. *Statistics and Computing, 27*(5), 1413–1432. DOI: https://doi.org/10.1007/s11222-016-9696-4
